# Co-benefits of nutrient management tailored to smallholder agriculture

**DOI:** 10.1016/j.gfs.2021.100570

**Published:** 2021-09

**Authors:** Pauline Chivenge, Kazuki Saito, Michelle Anne Bunquin, Sheetal Sharma, Achim Dobermann

**Affiliations:** a International Rice Research Institute (IRRI), DAPO Box 7777, Metro Manila, 1301, Philippines; b Africa Rice Center (AfricaRice), 01 B.P. 2551, Bouaké 01, Cote d’Ivoire; c International Rice Research Institute (IRRI), NASC Complex, Pusa, New Delhi, 110012, India; d International Fertilizer Association (IFA), 49 Avenue D’Iena, 75116, Paris, France; e Current Address: African Plant Nutrition Institute, UM6P Experimental Farm, Benguérir, 41350, Morocco

**Keywords:** Site-specific nutrient management, Sustainability, Digital tools, Nitrogen use efficiency, Rice, Wheat, Maize, Smallholder farmers, Fertilizer

## Abstract

Plant nutrition plays a central role in the global challenges to produce sufficient and nutritious food, lessen rural poverty, and reducing the environmental footprint of farming. Site-specific nutrient management (SSNM) provides field-specific solutions for smallholder farmers, potentially creating co-benefits of increased productivity and sustainability. Here we perform the first meta-analysis comparing SSNM with farmers’ fertilizer practice for maize, rice and wheat using 61 published papers across 11 countries. Relative to the farmer practice, across all crops SSNM increased grain yield by 12% and profitability by 15% with 10% less fertilizer nitrogen applied, thereby improving nitrogen use efficiency and reducing nitrogen pollution to the environment. Delivering it to millions of smallholder farmers requires use of digital decision support tools, but also policy incentives, links with financial and input supply services, and enhancing public-private partnerships.

## Highlights

• Meta-analysis was performed to compare SSNM with farmers' fertilizer practice (FFP).
• Data are from 61 published papers across 11 countries on maize, rice and wheat.
• Overall, 0.6 Mg ha^−1^ yield and US$ 140 ha^−1^ profit gains were obtained with SSNM.
• These benefits were achieved using 10% less N fertilizer than FFP.
• Agronomic N use efficiency was 40% greater with SSNM than FFP.

## Graphical abstract

**Figure:**
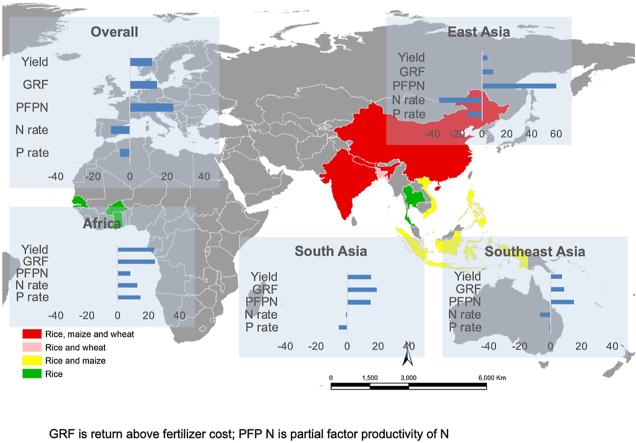

## 1 Introduction

Global needs to meet the growing food demand appear to conflict with pressures on land, biodiversity, environmental pollution and the changing climate (Foley et al., 2011). A new paradigm for plant nutrition needs to result in robust co-benefits for these seemingly contradicting goals, and it also needs to improve the nutritional quality of agricultural products (Cakmak, 2002). While inorganic fertilizers combined with high-yielding varieties, mechanization, irrigation and other inputs have contributed to sustained increases in crop yield and food security in the 20^th^ century (Cassman et al., 2003; Pingali, 2012; Lu and Tian, 2017), relying on blanket applications of fertilizer is inefficient and can cause excessive nutrient losses to the environment. Crop yield growth has been particularly slow in Sub-Saharan Africa and it has also slowed down in other world regions, suggesting that recent yield trends in agri-food systems are insufficient to meet the anticipated food demand from existing cropland (Ray et al., 2013). Plant nutrition plays a central role in producing sufficient and nutritious food, lessen rural poverty, and reducing the environmental footprint in the context of increasing competition for land and water (Bruulsema et al., 2020), and under more extreme weather conditions associated with climate change (Foley et al., 2011).

**Fig. 1:**
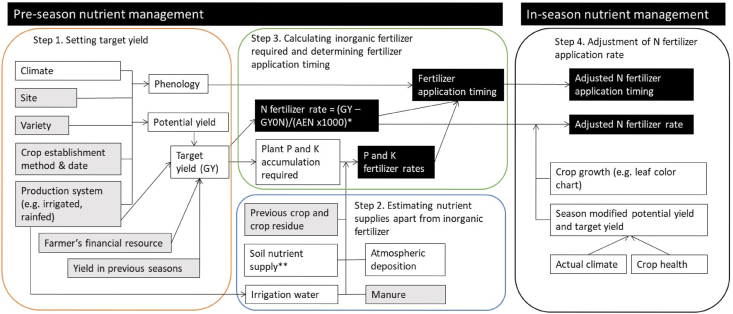
General framework and methodology for Site-Specific Nutrient Management (SSNM) recommendations. The actual implementation of this may vary, depending on types of decision support tools, available data sources, and additional algorithms that take into account major management practices. White boxes indicate input data previously collected in the field, reported by experts in this region, or calculated intermediate outputs; gray boxes indicate input information required from farmers' fields; black boxes indicate outputs.

One of the forward looking solutions to that is the Site-Specific Nutrient Management (SSNM) approach developed in the 1990s for smallholder cereal production systems in Asia (Dobermann et al., 2002, 2004; Buresh et al., 2010; Pampolino et al., 2012), which seeks to address large variability among farms and fields in their seasonal plant nutrient needs (Fig. 1) (Dobermann et al., 2002). The science behind SSNM was initially based on a stepwise model (Janssen et al., 1990) that allows calculating the required nitrogen (N), phosphorus (P), and potassium (K) amounts to attain a targeted yield, with additional rules for the timing and in-season adjustment of fertilizer applications (Fig. 1) (Dobermann et al., 2002; Pampolino et al., 2007; Wang et al., 2007; Buresh et al., 2010). SSNM thereby provided concrete decision advice to farmers on the rate, sources and time of fertilizer applications.

Over 25 years and through numerous national and international partnerships, SSNM evolved further in its underlying science, field methods and workflows, decision support tools and dissemination approaches, covering a growing number of crops, cropping systems, and countries in Asia and Africa. Growth stage or real-time approaches for in-season N management were developed, including optional use of diagnostic tools such as leaf color charts or chlorophyll meters (Fairhurst et al., 2007). Algorithms for P and K were improved (Buresh et al., 2010). Advances in information technology and mobile communications made it possible to generate tailored recommendations by using web- and smartphone-based apps such as Nutrient Expert (Pampolino et al., 2012; Xu et al., 2017a), Rice Crop Manager (Buresh et al., 2019; Sharma et al., 2019), and RiceAdvice (Arouna et al., 2021; Zossou et al., 2021). These tools were aimed at breaking the pervasive information and service asymmetry that is common in smallholder farming systems in the developing world, where there is a very low extension-to-farmer ratio, and where professional agricultural services such as soil testing are either not available, not affordable or not reliable.

Crops for which specific SSNM solutions have been developed so far include maize, rice, wheat, soybean, cassava, potatoes, and several others (Byju et al., 2016; Yang et al., 2017; Zhang et al., 2019). In China, the Nutrient Expert platform currently provides SSNM-based advice for 23 different crops, including fruits and vegetables. Many studies on SSNM were conducted in Asia, where fertilizer use is common in intensive farming, but SSNM was also introduced in Africa during the past 15 years, where low input cropping systems are still predominant, often with low yield, specific soil constraints, or degradation of soil health due to nutrient mining (Sanchez, 2002).

While the benefits of SSNM in cereal production systems have been demonstrated in numerous studies for rice (Dobermann et al., 2002; Peng et al., 2010; Saito et al., 2015), maize (Pasuquin et al., 2014; Shehu et al., 2019) and wheat (Khurana et al., 2008; Sapkota et al., 2014) in both Asia and Africa (Saito et al., 2015; Rurinda et al., 2020), previous studies have been limited to specific locations and conditions (Peng et al., 2010). A comprehensive and systematic synthesis across different crops and regions is lacking. Meta-analysis is a quantitative systematic review with statistical power to objectively synthesize results from multiple studies (Rosenburg et al., 2000; Gurevitch et al., 2018). However, critiques have cited flaws with their implementation, among them is publication bias, which emanates from selective publication of studies that reject the null hypothesis. This can lead to misleading conclusions, if analytical techniques to overcome publication bias are not used (Borenstein et al., 2009; Gurevitch et al., 2018).

Here we present the first global quantitative synthesis of the performance of SSNM and discuss the major lessons learned for maize, rice and wheat, which account for about 43% of the world's food calorie supply and consume about 51, 41 and 33% of global N, P and K fertilizers, respectively (Heffer et al., 2017). Using data from 61 published studies across 11 countries we performed a meta-analysis to compare grain yield, N use efficiency and profitability (gross return above fertilizer cost) of SSNM to the locally prevailing farmer fertilizer practice (FFP). Nitrogen use efficiency serves as a measure of the short-term balance between N used for grain production and that lost to the environment (Raun and Johnson, 1999; Cassman et al., 2002; Dobermann, 2007), and thus is an important indicator of environmental sustainability. In our meta-analysis we focused on two indices: agronomic N use efficiency (AEN), which represents additional grain yield per kg of N fertilizer applied, while the partial factor productivity of N (PFP N) represents grain yield produced per kg of N fertilizer applied. Whereas AEN is a measure of fertilizer N efficiency, PFP N is an important metric for farmers because it integrates the use efficiency of both indigenous (soil) and applied N resources.

## 2 Methodology

### 2.1 Database compilation

We compiled a database from 61 studies conducted in 11 countries (eight in Asia and three in Africa), published between 2001 and 2020 (SI Table 1; https://doi.org/10.7910/DVN/H23MVL). The literature search was conducted until June 12, 2020 using Web of Science, Science Direct and Google Scholar using the search terms: site-specific nutrient management (SSNM), SSNM rice, SSNM maize, SSNM wheat, SSNM cereals, SSNM vs farmers’ fertilizer practice (FFP). Studies included were peer reviewed journal publications, book chapters, or technical reports that explicitly compared SSNM and FFP in the same fields. Consequently, studies that compared SSNM to other treatments such as a no input control, blanket fertilizer recommendation, or soil test based recommendations were not included (Dogbe et al., 2015; Islam et al., 2018; Saito et al., 2019; Rurinda et al., 2020). Most of the studies reported in this study were conducted on-farm, and only five were conducted on-station. We only included studies that followed the generic SSNM approach described in at least steps 1 and 2 in Fig. 1 (Dobermann et al., 2002, Dobermann et al., 2004). Studies were also rejected if the experimental method was not clearly written and when factors other than fertilizer management differed between SSNM and FFP treatments. By and large, agronomic practices other than nutrient inputs were the same or similar in SSNM and FFP treatments. If data from the same experiments were reported in multiple publications, a paper having data with the most complete dataset was used. Of the reviewed studies, 66, 20, and 14% were on rice, wheat, and maize, respectively; all conducted in Asia, except three papers on rice in Africa (SI Table 1).

**Table 1:** Summary of grain yield, nitrogen (N) fertilizer application rate, number of N splits (No. N splits), agronomic use efficiency (AEN), partial factor of productivity of N (PFP N), phosphorus (P) and potassium (K) fertilizer application rate, and gross return on fertilizer (GRF) under prevailing farmer fertilizer practice (FFP) and site-specific nutrient management (SSNM) for rice, wheat and maize. The data are means ± standard error.

Crop/treatment	Grain yield (Mg ha^−1^)	N rate (kg ha^−1^)	No. N splits	AEN (kg grain kg^−1^ N)	PFP N (kg grain kg^−1^ N)	P rate (kg ha^−1^)	K rate (kg ha^−1^)	GRF (US$ ha^−1^)
**Overall**								
FFP	5.6 ± 0.1	143.3 ± 2.7	2.8 ± 0.1	12.1 ± 0.4	42.2 ± 0.9	25.0 ± 0.6	38.8 ± 1.8	1055 ± 23
SSNM	6.2 ± 0.1	125.4 ± 1.6	3.3 ± 0.1	16.9 ± 0.5	52.6 ± 1.2	24.0 ± 0.5	53.8 ± 1.2	1195 ± 22
**Rice**								
FFP	5.6 ± 0.1	131.6 ± 2.8	3.1 ± 0.1	12.2 ± 0.5	46.5 ± 1.3	22.8 ± 0.7	45.2 ± 2.1	1227 ± 24
SSNM	6.1 ± 0.1	113.4 ± 1.9	3.5 ± 0.1	16.8 ± 0.5	58.0 ± 1.6	22.7 ± 0.7	53.3 ± 1.7	1381 ± 24
**Wheat**								
FFP	4.3 ± 0.2	154.1 ± 6.6	2.4 ± 0.1	10.1 ± 0.8	27.8 ± 0.5	26.4 ± 1.2	16.0 ± 2.3	696 ± 37
SSNM	5.0 ± 0.2	137.8 ± 2.0	3.2 ± 0.1	14.1 ± 0.9	35.9 ± 1.1	26.8 ± 0.9	52.9 ± 2.1	837 ± 35
**Maize**								
FFP	7.4 ± 0.3	177.9 ± 7.3	2.1 ± 0.2	14.2 ± 1.6	44.2 ± 1.7	29.8 ± 2.1	48.2 ± 5.8	949 ± 44
SSNM	8.4 ± 0.3	160.7 ± 2.5	2.6 ± 0.1	21.3 ± 2.2	52.5 ± 1.8	24.5 ± 1.1	56.6 ± 2.8	1076 ± 41

*Where GY - GY0N is the increase in grain yield due to fertilizer N, which is the difference between the target yield (GY) (t/ha) and yield without N fertilizer (GY0N) (t/ha). The GY0N is the *N*-limited grain yield, which reflects the yield attainable from only soil plus other non-fertilizer sources of N, referred to as the soil N supply. AEN is the agronomic efficiency of fertilizer N (kg grain yield increase per kg of fertilizer N applied).

** Soil nutrient supply can be estimated with different methods, including nutrient omission trials or soil tests.

Agronomic N use efficiency (AEN) was reported in 27 studies, all of which had included a N omission treatment to enable calculation of AEN according to Equation (1). Partial factor of productivity of N (PFP N) was calculated for all studies that reported total N fertilizer added (Equation (2)).

(1) AEN = (GY_N_ (kg ha^−1^) – GY_0_ (kg ha^−1^))/N rate (kg N ha^−1^)

(2) PFP N = GY_N_ (kg ha^−1^)/ N rate (kg N ha^−1^)

Where GY_N_ is the grain yield in a treatment with N application.

GY_0_ is the grain yield in a treatment without N application.

Means for SSNM and FFP yields and AEN were retrieved from each study as well as the site characteristics including study location, study duration, soil properties and climatic conditions (SI Table 4). In few cases where yield and AEN data were only presented in figures, values were manually estimated from the figures. From the publications, categorical variables were assigned to cropping system, crop species, cropping season, ecosystem (irrigated/rainfed), variety (inbred/hybrid), residue management, crop establishment, type of SSNM recommendation, and type of trial. Crop yield categories were derived from the quartiles of the FFP grain yields of the whole dataset (SI Table 2). Values of FFP yield category below 1^st^ quartile were set as low, values between 1^st^ and 3^rd^ quartiles were set as medium, and values above 3^rd^ quartile were set as high. In some studies, farmers were considered as replicates within the same locality, e.g. within a village. Replicates in individual studies were variable but with highest numbers being 323, 510 and 701 for rice, maize and wheat, respectively. Due to high spatial variability even for neighboring fields, nutrient rates among the fields varied and were reported as a range. Thus, for this study, averages across the different replicates were obtained using the minimum and maximum values reported and were used as nutrient rates for reported grain yields. Urea was the most commonly used nitrogen fertilizer but diammonium phosphate (DAP), ammonium sulphate, and compound NPK fertilizers were also used. Muriate of potash (MOP) was the most commonly used potassium fertilizer while compound fertilizer was used in some cases. For phosphorus, single and triple super phosphate, DAP, and NPK fertilizers were used.

### 2.2 Economic analyses

We calculated three economic performance metrics: total fertilizer cost (TFC), gross return, and gross return above fertilizer cost (GRF). For the cost-benefit calculations, we assumed that fertilizer sources were the most common products recorded in the database: urea (46-0-0) for N, DAP (18-46-0) for N and K, and MOP (0-0-60) for K. Fertilizer prices were estimated from the 10-year average across countries listed in the database (indexmundi, 2020) and reported as per unit of nutrient. Values used were US$ 0.642 kg^−1^ N, US$ 2.151 kg^−1^ P, and US$ 0.633 kg^−1^ K. Farmgate prices of paddy rice, maize and wheat were derived from the trend of the market price for the past 25 years (indexmundi, 2020), with slight modifications based on consultations with an economist (Valerien Pede, IRRI personal communication). Prices used were US$ 0.25 kg^−1^ paddy rice, US$ 0.15 kg^−1^ maize and US$ 0.20 kg^−1^ wheat. Equations (3), (4), (5)) were used to calculate the economic parameters.

(3) TFC (US$ ha^−1^) = (pN × N_rate_) + (pP × P_rate_) + (pK × K_rate_)

(4) Gross return (US$ ha^−1^) = FGP × GY

(5) GRF (US$ ha^−1^) = Gross return – TFC

Where pN, pP, pK = prices of N, P and K fertilizers, respectively (US $ kg^−1^)

N_rate_, P_rate_, K_rate_ = amount of N, P and K applied (kg ha^−1^)

FGP = farmgate price of paddy rice, maize or wheat (US$ kg^−1^)

GY = grain yield of paddy rice, maize and wheat (kg ha^−1^)

### 2.3 Meta-analysis

Grain yield, N fertilizer rates, number of N splits, AEN, PFPN and economic parameters were analyzed using MetaWin 2.1 software (Rosenburg et al., 2000). The natural log of the response ratio (the ratio of SSNM to FFP) was used as the effect size in our meta-analysis in Equation (6). In most of the studies, the within-study variance measures for mean yields were not reported. Thus, individual observations were weighted by replication (Adams et al., 1997), as shown in Equation (7).

(6) Effect size = ln (Xe/Xc)

where Xe is the mean data for SSNM while Xc is the mean data for FFP, which was used as the control.

(7) Weights = (N_SSNM_ x N_FFP_)/(N_SSNM_ + N_FFP_)

where N_SSNM_ and N_FFP_ are the number of replicates for SSNM and FFP treatments, respectively.

The potential for publication bias in the literature was tested to determine the impact of effect size of large studies as compared with small studies (Borenstein et al., 2009). This was done by constructing a funnel plot with the effect size on the x-axis vs the variance on the y-axis. The plot resembled a funnel where large studies were presented toward the top of the graph and generally clustered around the mean effect size while smaller studies appeared toward the bottom of the graph since they have more sampling error variation in effect sizes. Our study acknowledged the presence of publication bias since the studies resembled a funnel plot which is asymmetrical and had missing studies in the middle and near the bottom of the plot. We used a weighted analysis of data (Equation (7)) to reduce bias of magnifying the influence of small studies.

Mean effect size of each categorical variable was calculated with bias corrected 95% confidence interval generated by the bootstrapping procedure in MetaWin (using 4999 iterations). Differences for each variable between SSNM and FFP were considered significant if the 95% confidence interval did not overlap zero. For convenience in interpretation, all results were reported as percentage change in yield, N fertilizer rates, number of N splits, AEN, PFP N, TFC, gross return and GRF for SSNM relative to FFP.

## 3 Results

### 3.1 Overall responses to SSNM

**Fig. 2:**
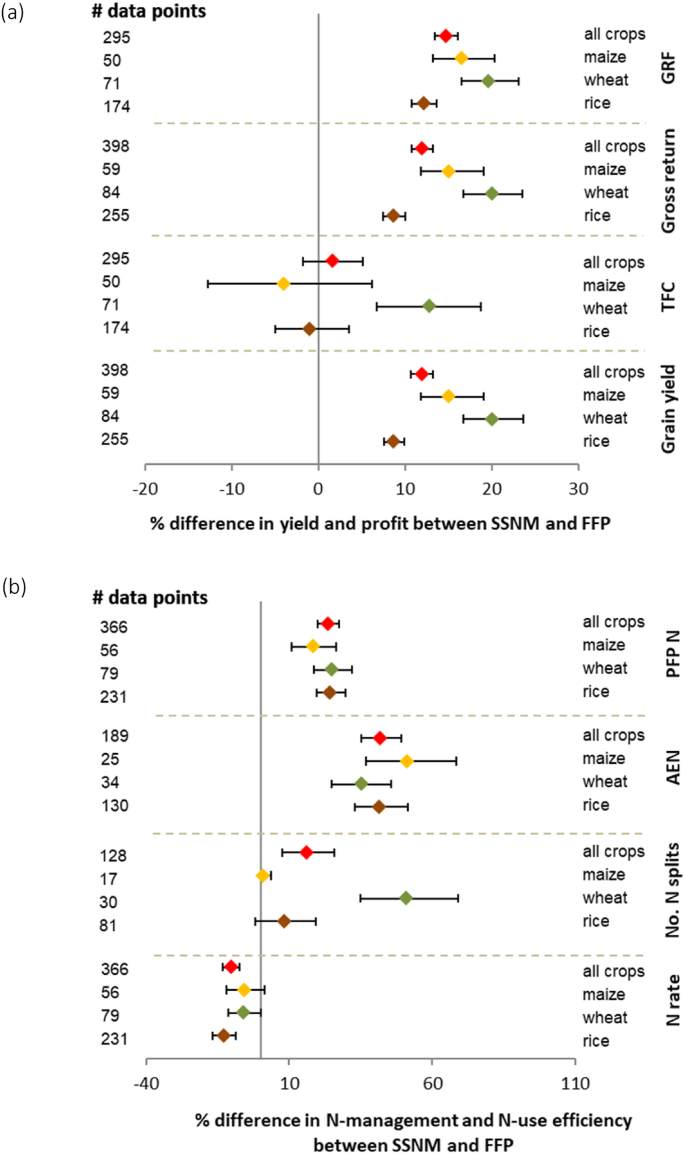
a) Grain yield, total fertilizer cost (TFC), gross return and gross return above fertilizer cost (GRF), and b) N use efficiency (agronomic N use efficiency; AEN, partial factor of productivity; PFP N) responses to site-specific nutrient management (SSNM) compared to the farmer fertilizer practice (FFP) for rice, wheat and maize. Responses are expressed as mean response percentage with 95% confidence intervals represented by error bars. Numbers of effect size comparisons are given as # of data points.

Across all three crops, we observed significant co-benefits of SSNM in terms of greater grain yield, profit and N use efficiencies than FFP (Table 1; Fig. 2). On average, yield gains were 0.6 Mg ha^−1^ (12%). Gross return increased by 12%, whereas gross return above fertilizer cost was 15% higher with SSNM compared to FFP. On average farmers’ average profit increased by US$ 140 ha^−1^. This was achieved with about 18 kg N ha^−1^ less N fertilizer, which resulted in significantly greater AEN under SSNM than FFP (17 vs 12 kg grain kg^−1^ N applied) and PFP N (54 vs 43 kg grain kg^−1^ N applied). Besides field-specific tailoring of N amounts, the increased N use efficiency in SSNM was also due to differences in frequencies and timing of N fertilizer application, and P and K fertilizer application rates between the two fertilizer management approaches. SSNM involved more splits of *N*-fertilizer than FFP (3.1 vs 2.6 times), resulting in better congruence of N supply with key periods of crop growth and demand (Cassman et al., 2002). Balanced P and K fertilizer application based on field-specific nutrient requirements and input-output budgets in SSNM enhances uptake and utilization of applied N (Janssen et al., 1990). On average, there was no difference in P fertilizer application rate between SSNM and FFP, whereas SSNM included higher K fertilizer application rates (Table 1). The typically observed 40–50% relative increases in AEN with SSNM suggests reduced N losses to the environment, given that N use efficiency represents the balanced between N uptake and N lost (Raun and Johnson, 1999; Cassman et al., 2002; Dobermann, 2007). This is an important contribution to improving N input-output budgets in global food production systems, particularly in regions where N use efficiency has decreased over time with increased fertilizer use, such as East Asia (Ladha et al., 2016).

### 3.2 Differential SSNM responses by crop

**Fig. 3:**
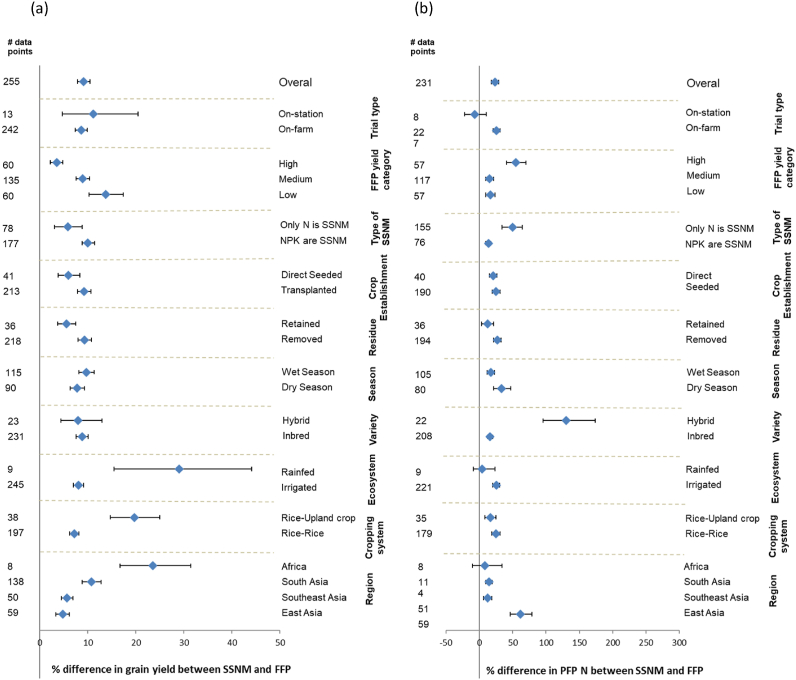
Rice a) grain yield, and b) N use efficiency (partial factor of productivity; PFP N) responses to site-specific nutrient management compared to the farmer fertilizer practice (FFP) for different categories (geographical region, cropping system, variety, cropping season, ecosystem type, residue management, crop establishment method, decision support tool, yield category for FFP, and type of trial). Responses are expressed as mean response percentage with 95% confidence intervals represented by error bars. Numbers of effect size comparisons are given as # of data points.

**Fig. 4:**
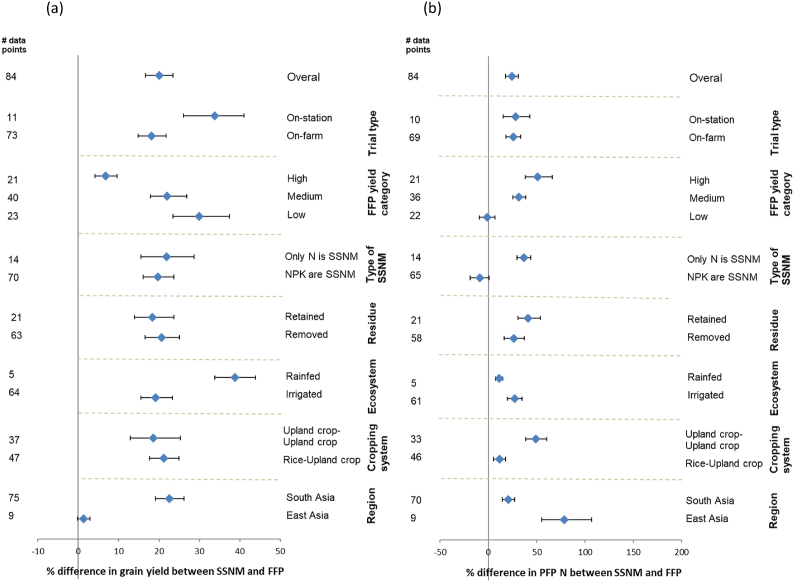
Wheat a) grain yield, and b) N use efficiency (partial factor of productivity; PFP N) responses to site-specific nutrient management compared to the farmer fertilizer practice (FFP) for different categories (cropping system, cropping ecosystem type, residue management, crop establishment method, decision support tool, yield category for FFP, and type of trial). Responses are expressed as mean response percentage with 95% confidence intervals represented by error bars. Numbers of effect size comparisons are given as # of data points.

**Fig. 5:**
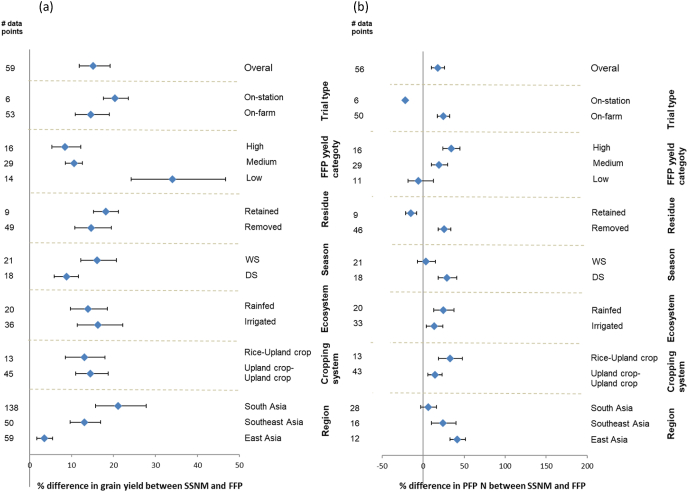
Maize a) grain yield, and b) N use efficiency (partial factor of productivity; PFP N) responses to site-specific nutrient management compared to the farmer fertilizer practice (FFP) for different categories (cropping system, cropping ecosystem type, residue management, crop establishment method, decision support tool, yield category for FFP, and type of trial). Responses are expressed as mean response percentage with 95% confidence intervals represented by error bars. Numbers of effect size comparisons are given as # of data points.

While the total fertilizer cost was generally comparable between SSNM and FFP for rice and maize, wheat had 13% higher fertilizer cost for SSNM (Fig. 2), but also a higher increase in grain yield and profit with SSNM, followed by maize. A correlation analysis indicated that increases in grain yield and gross return above fertilizer cost under SSNM were strongly correlated across all crops (SI Table 3). Hence for a breakdown analysis by crop we only discuss yield and PFP N, as the N use efficiency index. Surprisingly, grain yield and PFP N were not correlated for maize and wheat, and the relationship was weak for rice too (Data not shown). Furthermore, in regions such as China, where grain yield in FFP was high due to very high fertilizer rates, increased grain yields were typically associated with substantial reductions in N application rate in SSNM, and N use efficiency was greatly improved across all crops (Fig. 3, Fig. 4, Fig. 5) (Pampolino et al., 2007; Peng et al., 2010; Xu et al., 2017a). Some studies have shown that increased yield and PFP N with SSNM were associated with a reduction in insect and disease damage and improved lodging resistance of rice crop (Sta Cruz et al., 2007; Peng et al., 2010). Surplus N causes excessive vegetative growth, which makes crops prone to lodging, and pest and disease attack. Large yield gains with SSNM were particularly observed when FFP yield was low, including in lower-yielding regions such as Africa and South Asia (Fig. 3, Fig. 4, Fig. 5). For example, rice yield increases through SSNM were 24% in Africa and 10% in South Asia, but the difference in N use efficiency between the SSNM and FFP treatments was smaller in these regions than in China. That is because N fertilizer rates in SSNM were often higher than in FFP in the low-yielding regions. These contrasting results illustrate a very robust performance of the field-specific tailoring of nutrient management in diverse production situations and environments, showing that achieving high yields and profit in combination with lowering nutrient losses are not elusive or conflicting goals.

### 3.3 Management and environmental effects

We also evaluated the effect of other agronomic practices on the performance of SSNM. Greater yield response to SSNM compared to FFP was observed when rice was rotated with an upland crop compared to rice grown continuously (19.7 vs 7.6%; Fig. 3). This was not the case for maize and wheat yield (Fig. 4, Fig. 5). Nitrogen use efficiency showed inconsistent results between maize and wheat. Growing rice before maize resulted in better response of maize to SSNM in terms of N use efficiency, whereas this was opposite for wheat. Crop residue management did not influence grain yield and N use efficiency responses to SSNM among the three crops, except for maize, in which PFP N was 15% lower in SSNM than FFP when residue was retained. Different variety choices (inbreds vs. hybrids) or different crop establishment methods for rice, had no impact on yield, but N use efficiency was greater under SSNM for hybrid rice than with inbred cultivars (Fig. 3), which may reflect a more vigorous growth and N uptake behavior of hybrids.

Yield gains with SSNM tended to be greater in the wet than dry season for rice and maize, yet N use efficiency benefits tended to be greater in the dry season, with no differences between SSNM and FFP for maize. Similarly, yield gain with SSNM tended to be greater for studies conducted on-station than on-farm, (Fig. 3, Fig. 4, Fig. 5). Most of the studies for rice and wheat were conducted under irrigated conditions, as SSNM had initial focus on favorable growing environments. However, our analysis shows that SSNM improved both grain yield and N use efficiency of the three crops under rainfed as well as irrigated conditions (Haefele and Konboon, 2009; Banayo et al., 2018). Grain yield gain and N use efficiency benefit with SSNM over FFP tended to be greater for transplanted than direct-seeded rice (Fig. 3). While only 12 studies evaluated SSNM under direct-seeded rice, our results suggest the need to fine tune SSNM recommendations for these systems, which are increasingly replacing transplanted rice in response to water and labor scarcity (Cabangon et al., 2002; Pampolino et al., 2007). Early studies showed small rice yield gain with SSNM for direct-seeded rice and cited opportunities for SSNM to improve as an integrated crop management approach by including the adaptation to local crop establishment methods, water and weed management (Rao et al., 2007; Liu et al., 2013).

### 3.4 Economic and environmental impact assessment

Our meta-analysis mainly focused on researcher-managed, on-farm experiments, and we recognize the problems of extrapolating plot-scale research results to whole field, farm or landscape scale (Dobermann, 2012). When farmers adopt SSNM on a larger scale, the benefits from SSNM might differ from what was observed in field experiments, as farmers may adjust the specific SSNM recommendations and management practices to their conditions and belief systems. Additionally, full adoption of recommendations can be affected by time and resource constraints. However, a few available impact assessment studies of SSNM with thousands of smallholder farmers have also shown grain yield improvements of 2–17% and profitability increases of 4–48% (SI Table 4), which is comparable to the results of our meta-analysis.

We could not directly assess environmental impacts of SSNM in a systematic manner because of the paucity of data. However, several specific studies indicate that there are substantial benefits beyond the metrics we have used, i.e. additional co-benefits for soil health, greenhouse gas emissions, and other forms of nutrient losses, such as leaching and runoff (Pampolino et al., 2007; Wang et al., 2007; Liang et al., 2013; An et al., 2015). Environmental effects assessment in Northeast China demonstrated that SSNM reduced reactive N losses and greenhouse gas emissions by 46.9% and 37.2% for maize, respectively, and 10.1% and 6.6% for rice, respectively (Wang et al., 2020). Reduced N losses were associated with increased N use efficiency and N uptake. Similarly, Sapkota et al. (2014) showed reduced greenhouse gas emissions with SSNM compared to FFP in a wheat cropping system under conservation agriculture in West India. Adoption of SSNM was shown to improve physical, chemical and biological soil properties in a maize-wheat-mung bean rotation system with conservation agriculture in India compared to the no fertilizer treatment (Parihar et al., 2020). Zhang et al. (2018) observed a 55% reduction in nitrous oxide emissions with SSNM relative to FFP, associated with 70% greater AEN under SSNM in a four year study on wheat in 315 field studies in North-Central China. Clearly, SSNM can address these environmental challenges, while closing crop yield gaps and enhancing profit, thus meeting many sustainable intensification target (Tilman et al., 2011).

## 4 Discussion

Our study acknowledged the presence of publication bias and employed analytical tools to reduce the bias (Borenstein et al., 2009). We applied strict selection criteria for inclusion or exclusion of literature, which left us with a seemingly small number of studies (61), but this allowed us to conduct a more consistent and objective meta-analysis, with close to 400 paired comparisons for grain yield. We also acknowledge that the dataset used in our study was geographically biased, with very few studies coming from Africa, highlighting the paucity of data in the region. Nonetheless, we know of no other agronomic intervention that has increased crop yield, profitability, and N use efficiency across three cereal crops and geographies in such a robust manner. This suggests that SSNM – through field-specific tailoring of fertilizer applications -- is a highly effective management strategy that maximizes positive outcomes, contributing to food security attainment with economic and environmental benefits. While we could not directly evaluate environmental impacts in our meta-analysis, we clearly show increased N use efficiency with an average 10% reduction of N fertilizer rate N under SSNM compared to FFP (Table 1). The surplus reactive N under FFP is prone to losses. A 10% reduction in N fertilizer use in major food systems that consume 51% of global N fertilizer (Heffer et al., 2017), can substantially reduce excess reactive N and promote economic and environmental prosperity. Our study also showed higher N use efficiency benefits with reduced N fertilizer rates in regions like China where farmers use high amounts of N fertilizers (Xu et al., 2017a).

Despite the clear co-benefits of SSNM, it has proven to be difficult to scale SSNM up and achieve widespread adoption by millions of smallholder farmers. In recent years, with advances in information technology, SSNM has been disseminated to extension workers and farmers through a variety of digital decision support tools as well as other means. For example, Rice Crop Manager (https://phapps.irri.org/ph/rcm/) generated 2.66 millions fertilizer recommendations in the Philippines (2013 to early 2021) and 250,000 in India (http://webapps.irri.org/in/od/rcm/, http://webapps.irri.org/in/br/cmrs/; 2017 to early 2021), whereas in West African countries (2014–2020) RiceAdvice (https://www.riceadvice.info/en/) generated 100,000 recommendations. The high number of recommendations in the Philippines reflects stronger support by the Philippine government, with Rice Crop Manager recently becoming part of a country-wide agriculture digital advisory service (https://rcm.da.gov.ph/). Uptake of the SSNM recommendations in the Philippines has reached about 30%, but there are still a myriad of constraints that farmers face.

In China, a Nutrient Expert software platform (http://www.nutrientexpert.cn/) of web and smartphone apps, including social media (WeChat), is being used to develop, validate and disseminate SSNM solutions for 23 different crops, including grain crops, cash crops, vegetables, and fruits. The platform has about 10,000 registered users who make recommendation for farmers (averaging at least 100 recommendations in recent years per user), and collaboration with fertilizer companies is evolving (He Ping, Chinese Academy of Agricultural Sciences, personal communication). In contrast, in Africa dissemination of Nutrient Expert has been limited to maize and requires further development of suitable business models to reach more farmers (Shamie Zingore, African Plant Nutrition Institute, personal communication).

Providing customized and actionable agricultural information via digital technologies has benefits that likely exceed the cost of information generation and transmission by far, but realizing that potential requires good feedback mechanisms to enable co-design, rigorous testing and continuous improvement (Fabregas et al., 2019). Across geographies and crops, lessons learned so far from dissemination of SSNM show that limited resources, apps that are too complex to use, low level of literacy and lack of ICT skills, limited access to smartphones or tablets and internet, insufficient integration of other agronomically relevant information and lacking integration with financial and input supply services are common constraints that need to be overcome. There is also potential to out-scale SSNM more quickly. For example, key elements of SSNM have also been incorporated into a new global standard for sustainable rice production (SRP, 2019), and in China, geospatial (regional) approaches were used as a first step before moving down to field-scale tailoring of advice (Xu et al., 2017b; Ding et al., 2020). Overall, however, scaling SSNM up and out will require much bigger public and private sector investments than made so far to create a multitude of dissemination channels, especially in areas where public extension services are currently not strong (Zossou et al., 2021).

Policies that support the adoption of innovative, evidence-based practices for better fertilizer management can provide a better enabling environment than policies that focus on fertilizer subsidies per se. Most important, however, will be the integration of SSNM in sustainability-driven business models of all actors in the crop production chain, from advisors and input suppliers to farmers, processors and retailers. These potential enabling actions for scaling SSNM are not well studied and can be addressed by interdisciplinary teams (Arouna et al., 2021). Developing landscape-scale recommendations, public-private partnerships, integrating the tools with existing digital platforms and agro-advisory services can lead to increased outreach of SSNM benefits to a large numbers of farmers (unpublished data).Finally, it is noted that recently, Excellence in initiative (EiA 2030) was established by CGIAR research centers (AfricaRice, CIAT, CIMMYT, CIP, ICARDA, ICRAF, ICRISAT, IFPRI, IITA, and IRRI), responds to demand from renewed public and private sector for scalable agronomic solutions, and co-develops and co-validates agronomy at scale solutions, operating in all regions of the Global South. This new approach is different from research-driven solution development like SSNM and its digital decision support tools available now. We expect that full or key SSNM elements could be integrated into tailor-made tools co-developed with different partners for reaching millions of farmers.

## 5 Conclusion

Results from our meta-analysis clearly show that SSNM creates co-benefits, with greater yields, profit, and N use efficiency when compared with the farmer fertilizer practice for maize, rice and wheat. SSNM reduces N and P fertilizer rates in most cases, except in situations where nutrients are mined such as in Africa, and is essential component of agronomic solutions for sustainable crop production. These benefits were realized across geographies and under variable conditions, indicating a very robust performance in widely varying environments and management options, although rigorous testing and continuous improvement is needed. Digital tools have been developed for widespread dissemination of SSNM recommendations, but uptake by farmers has remained low. Reaching millions of farmers can be achieved through integration of policy incentives, financial and input supply services, and improved knowledge exchange among extension, public and private partners.

## Declaration of competing interest

The authors declare that they have no known competing financial interests or personal relationships that could have appeared to influence the work reported in this paper.

## Data Availability

Data can be retrieved from DOI: https://doi.org/10.7910/DVN/H23MVL.
