# Supplementary material for: Co-benefits of nutrient management tailored to smallholder agriculture
Source: Glob Food Sec. 2021 Sep;30:100570. doi: 10.1016/j.gfs.2021.100570 (PMC8429639; doi:10.1016/j.gfs.2021.100570)
Supplement: Multimedia component 1 [file mmc1.docx]

**Supplementary information Table 1: Data sets included in the database, with countries, cropping systems, crop type, seasons, number of replications, number of nitrogen (N) splits, mean N, phosphorus (P) and potassium (K) fertilizer rates, mean grain yield, agronomic efficiency of N (AEN), partial factor of productivity N (PFP N), total fertilizer cost (TFC), gross return, and gross return above fertilizer cost (GRF) under Site-Specific Nutrient Management (SSNM) and Farmers’ Fertilizer Practice (FFP).**

| **Ref** | **Country** | **Cropping**  **system^ξ^** | **Crop** | **Season^ϑ^** | **Rep** | **No. of N splits** | | **N rate**  **(kg ha^-1^)** | | **P rate**  **(kg ha^-1^)** | | **K rate**  **(kg ha^-1^)** | | **Grain yield**  **(kg ha^-1^)** | | **AEN**  **(kg grain kg^-1^ N)** | | **PFP N**  **(kg grain kg^-1^ N)** | | **TFC**  **(USD ha^-1^)** | | **Gross return**  **(USD ha^-1^)** | | **GRF**  **(USD ha^-1^)** | |
| --- | --- | --- | --- | --- | --- | --- | --- | --- | --- | --- | --- | --- | --- | --- | --- | --- | --- | --- | --- | --- | --- | --- | --- | --- | --- |
|  |  |  |  |  |  | **SSNM** | **FFP** | **SSNM** | **FFP** | **SSNM** | **FFP** | **SSNM** | **FFP** | **SSNM** | **FFP** | **SSNM** | **FFP** | **SSNM** | **FFP** | **SSNM** | **FFP** | **SSNM** | **FFP** | **SSNM** | **FFP** |
| ^1^ | Indonesia | R-R | Rice | DS | 20 | 3 |  | 123.5 | 141.0 | 19.3 | 6.5 | 73.0 | 4.8 | 3.9 | 3.6 | 10.3 | 8.4 | 32.0 | 25.7 | 156 | 104 | 975 | 906 | 819 | 802 |
|  | Indonesia | R-R | Rice | WS | 20 | 4 |  | 87.5 | 100.3 | 20.5 | 8.5 | 40.0 | 4.3 | 5.1 | 4.9 | 15.0 | 9.9 | 58.2 | 49.4 | 114 | 80 | 1275 | 1231 | 1161 | 1151 |
| ^2^ | Ghana | R-R | Rice | DS and  WS | 50 |  |  | 126.0 | 151.0 | 24.4 | 22.9 | 46.5 | 43.6 | 4.9 | 4.3 |  |  | 38.9 | 28.5 | 149 | 161 | 1225 | 1075 | 1076 | 914 |
| ^3^ | Bangladesh | R-R | Rice | DS | 23 | 5 | 3 | 137.5 | 164.3 | 29.4 | 29.4 | 43.4 | 43.4 | 6.0 | 5.5 | 21.5 | 15.9 | 43.4 | 33.9 | 162 | 179 | 1491 | 1378 | 1328 | 1199 |
|  | Bangladesh | R-R | Rice | WS | 36 | 5 | 3 | 123.4 | 135.4 | 22.9 | 22.5 | 32.4 | 32.4 | 4.5 | 3.9 | 16.8 | 11.1 | 36.9 | 30.5 | 136 | 143 | 1113 | 966 | 977 | 823 |
| ^4^ | Bangladesh | R-U | Rice | WS | 10 | 5 | 4 | 116.7 | 149.3 | 25.2 | 30.3 | 40.7 | 46.3 | 4.6 | 4.0 | 16.8 | 10.3 | 39.3 | 26.8 | 140 | 173 | 1138 | 1000 | 997 | 827 |
|  | Bangladesh | R-U | Wheat | DS | 10 | 5 | 3 | 120.0 | 91.0 | 30.2 | 24.4 | 26.2 | 27.1 | 2.8 | 2.3 | 14.0 | 11.1 | 23.6 | 26.1 | 141 | 114 | 567 | 463 | 421 | 349 |
| ^5^ | Philippines | R-R | Rice | WS | 34 | 4 | 5 | 81.5 | 96.5 | 10.1 | 11.4 | 21.2 | 18.9 | 4.5 | 4.2 |  |  | 56.3 | 44.5 | 82 | 92 | 1134 | 1057 | 1053 | 965 |
| ^6^ | India | U-U | Wheat | DS | 100 |  |  | 123.0 | 117.0 | 27.0 | 23.5 | 68.9 | 0.0 | 5.2 | 3.8 |  |  | 42.5 | 32.2 | 165 | 112 | 1045 | 755 | 880 | 642 |
| ^7^ | India |  | Rice |  | 165 |  |  | 98.8 | 131.6 |  |  |  |  | 6.5 | 6.4 | 23.0 | 17.8 | 66.9 | 48.5 |  |  | 1617 | 1596 |  |  |
| ^8^ | India | R-U | Rice |  | 5 |  |  | 200.0 | 120.0 | 43.6 | 13.1 | 83.0 | 24.9 | 5.5 | 3.7 |  |  | 27.6 | 30.7 | 250 | 113 | 1380 | 922 | 1130 | 808 |
|  | India | R-U | Wheat |  | 5 |  |  | 125.0 | 100.0 | 43.6 | 21.8 | 41.5 | 24.9 | 3.7 | 2.6 |  |  | 29.3 | 26.4 | 175 | 114 | 733 | 528 | 557 | 414 |
| ^9^ | Nepal | R-U | Rice | WS | 4 |  |  |  |  |  |  |  |  | 5.1 | 4.0 |  |  |  |  |  |  | 1285 | 1005 |  |  |
| ^10^ | China | U-U | Wheat | DS | 100 | 3 |  | 140.0 | 279.8 | 28.9 | 52.3 | 55.8 | 45.9 | 7.9 | 7.9 | 9.0 | 5.4 | 56.9 | 28.8 | 171 | 291 | 1585 | 1578 | 1414 | 1286 |
| ^11^ | Nepal | U-U | Maize |  | 20 | 3 |  | 150.0 |  | 39.2 |  | 83.0 |  | 7.0 | 4.0 |  |  | 46.4 |  | 211 |  | 1045 | 595 | 834 |  |
|  | Nepal | U-U | Wheat |  | 20 | 3 |  | 170.0 |  | 39.2 |  | 66.4 |  | 6.0 | 3.3 |  |  | 35.5 |  | 213 |  | 1208 | 658 | 994 |  |
| ^12^ | India | U-U | Wheat | DS | 26 | 3 |  | 139.8 | 148.7 | 25.1 | 25.1 | 64.6 | 14.5 | 5.2 | 4.4 |  |  | 37.3 | 29.8 | 170 | 144 | 1035 | 883 | 864 | 739 |
| ^13^ | Philippines | R-R | Rice | DS | 27 | 5 | 3 | 132.0 | 129.5 | 20.5 | 15.4 | 55.5 | 26.5 | 5.7 | 5.1 | 16.3 | 13.0 | 43.4 | 39.5 | 152 | 124 | 1431 | 1275 | 1279 | 1151 |
|  | Philippines | R-R | Rice | WS | 27 | 5 | 3 | 88.0 | 85.3 | 17.5 | 14.5 | 41.5 | 19.3 | 4.7 | 4.3 | 13.0 | 11.5 | 53.2 | 50.5 | 110 | 90 | 1169 | 1075 | 1058 | 985 |
| ^14^ | Nepal | R-U | Rice | DS | 12 |  |  |  |  |  |  |  |  | 5.5 | 4.4 |  |  |  |  |  |  | 1365 | 1108 |  |  |
| ^15^ | China | R-R | Rice |  | 108 | 5 | 5 | 141.5 | 177.0 |  |  |  |  | 6.1 | 5.9 |  |  | 43.2 | 33.4 |  |  | 1525 | 1475 |  |  |
| **Ref** | **Country** | **Cropping**  **system^ξ^** | **Crop** | **Season^ϑ^** | **Rep** | **No. of N splits** | | **N rate**  **(kg ha^-1^)** | | **P rate**  **(kg ha^-1^)** | | **K rate**  **(kg ha^-1^)** | | **Grain yield**  **(kg ha^-1^)** | | **AEN**  **(kg grain kg^-1^ N)** | | **PFP N**  **(kg grain kg^-1^ N)** | | **TFC**  **(USD ha^-1^)** | | **Gross return**  **(USD ha^-1^)** | | **GRF**  **(USD ha^-1^)** | |
|  |  |  |  |  |  | **SSNM** | **FFP** | **SSNM** | **FFP** | **SSNM** | **FFP** | **SSNM** | **FFP** | **SSNM** | **FFP** | **SSNM** | **FFP** | **SSNM** | **FFP** | **SSNM** | **FFP** | **SSNM** | **FFP** | **SSNM** | **FFP** |
| ^16^ | India | R-R | Rice | DS | 98 |  |  | 106.4 | 133.6 |  |  |  |  | 4.1 | 4.1 |  |  | 38.5 | 30.2 |  |  | 1032 | 1016 |  |  |
|  | India | R-R | Rice | WS | 98 |  |  | 100.4 | 121.2 |  |  |  |  | 3.5 | 3.4 |  |  | 34.6 | 28.2 |  |  | 868 | 855 |  |  |
| ^17^ | India | U-U | Maize | WS | 3 | 3 | 3 | 157.0 | 138.0 | 20.9 | 25.3 | 69.7 |  | 7.8 | 7.0 |  |  | 49.9 | 50.4 | 178 |  | 1175 | 1043 | 997 |  |
|  | India | U-U | Wheat | DS | 3 | 2 | 2 | 132.5 | 195.0 | 22.9 | 25.3 | 66.8 |  | 5.5 | 4.7 |  |  | 41.2 | 24.0 | 164 |  | 1093 | 937 | 929 |  |
| ^18^ | Nepal |  | Maize |  | 20 |  |  |  |  |  |  |  |  | 9.2 | 4.9 |  |  |  |  |  |  | 1383 | 741 |  |  |
| ^19^ | Vietnam | R-R | Rice | DS | 10 |  |  | 99.8 | 108.7 | 18.9 | 21.6 |  | 42.9 | 6.1 | 6.1 | 15.1 | 13.7 | 61.0 | 55.7 |  | 131 | 1523 | 1515 |  | 1384 |
|  | Vietnam | R-R | Rice | WS | 10 |  |  | 92.0 | 102.8 | 20.1 | 20.3 |  | 34.7 | 5.2 | 5.0 | 15.2 | 12.3 | 56.0 | 48.8 |  | 120 | 1288 | 1248 |  | 1127 |
| ^20^ | India | R-U | Rice | WS | 11 | 3 |  | 135.8 | 147.5 | 11.2 | 2.9 | 29.5 | 0.0 | 6.0 | 5.1 | 16.6 | 9.4 | 44.3 | 35.4 | 124 | 99 | 1500 | 1279 | 1376 | 1180 |
| ^21^ | India | R-U | Wheat | DS | 11 | 3 | 2 | 128.3 | 147.5 | 13.0 | 15.0 | 29.5 | 0.0 | 4.8 | 4.2 | 13.6 | 8.4 | 37.4 | 28.6 | 122 | 118 | 953 | 843 | 832 | 725 |
| ^22^ | India | R-U | Rice | WS | 56 | 4 | 4 | 137.0 | 148.0 |  |  |  |  | 6.0 | 5.1 | 16.0 | 9.5 | 43.8 | 34.1 |  |  | 1500 | 1263 |  |  |
|  | India | R-U | Wheat | DS | 56 |  |  | 128.0 | 143.0 |  |  |  |  | 4.9 | 4.4 | 14.0 | 9.5 | 38.3 | 30.8 |  |  | 980 | 880 |  |  |
| ^23^ | India | R-U | Wheat | DS | 29 | 3 |  | 156.0 | 170.0 | 23.1 | 25.3 | 69.7 | 0.8 | 5.3 | 4.7 |  |  | 34.0 | 27.4 | 181 | 150 | 1061 | 933 | 880 | 784 |
| ^24^ | India | U-U | Maize |  | 510 |  |  | 130.0 | 138.0 | 16.1 | 10.0 | 46.5 | 0.0 | 8.4 | 7.8 |  |  | 64.6 | 56.5 | 138 | 104 | 1260 | 1170 | 1122 | 1066 |
| ^25^ | India | R-U | Rice | WS | 323 |  |  | 111.0 | 85.0 | 14.4 | 23.1 | 40.7 | 39.0 | 5.8 | 4.6 |  |  | 52.1 | 54.4 | 120 | 116 | 1446 | 1157 | 1326 | 1041 |
| ^26^ | Nepal | R-U | Rice | WS | 7 |  |  | 95.8 | 52.5 | 18.7 | 11.3 | 59.8 | 6.9 | 6.4 | 4.6 |  |  | 66.3 | 88.0 | 129 | 56 | 1588 | 1155 | 1459 | 1099 |
|  | Nepal | R-U | Rice | WS | 7 |  |  | 100.0 | 52.5 | 18.7 | 11.3 | 59.8 | 6.9 | 6.7 | 4.6 |  |  | 66.6 | 88.0 | 132 | 56 | 1665 | 1155 | 1533 | 1099 |
| ^27^ | India | R-R | Rice | DS | 25 | 4 |  | 129.4 | 93.2 | 22.1 | 22.9 | 75.1 | 32.7 | 6.6 | 5.8 | 15.2 | 13.6 | 50.6 | 66.0 | 165 | 117 | 1638 | 1447 | 1472 | 1330 |
|  | India | R-R | Rice | WS | 25 |  |  | 128.3 | 103.7 | 22.1 | 20.1 | 75.1 | 36.6 | 5.5 | 4.9 | 15.2 | 13.6 | 42.8 | 47.3 | 165 | 122 | 1373 | 1228 | 1208 | 1107 |
| ^28^ | India | R-R | Rice | DS | 5 | 4 | 4 | 118.5 | 113.0 | 13.8 | 21.5 | 67.0 | 37.5 | 6.4 | 6.0 |  |  | 54.2 | 53.2 | 140 | 130 | 1606 | 1500 | 1466 | 1370 |
|  | India | R-R | Rice | WS | 5 | 4 | 3 | 130.5 | 116.5 | 13.8 | 17.5 | 65.0 | 34.5 | 6.4 | 6.0 |  |  | 49.4 | 52.8 | 147 | 124 | 1606 | 1500 | 1460 | 1376 |
|  | Philippines | R-R | Rice | DS | 5 | 3 | 3 | 119.0 | 156.5 | 15.0 | 18.0 | 54.0 | 36.5 | 5.2 | 4.9 |  |  | 43.7 | 31.0 | 134 | 152 | 1300 | 1213 | 1166 | 1060 |
|  | Philippines | R-R | Rice | WS | 5 | 5 | 2 | 106.0 | 93.0 | 9.0 | 15.5 | 42.0 | 24.5 | 5.2 | 4.9 |  |  | 49.1 | 52.2 | 109 | 100 | 1300 | 1213 | 1191 | 1113 |
|  | Vietnam | R-R | Rice | DS | 5 | 3 | 3 | 99.3 | 112.7 | 20.3 | 19.0 | 42.0 | 41.3 | 4.9 | 4.6 |  |  | 49.5 | 41.0 | 123 | 129 | 1229 | 1146 | 1107 | 1017 |
|  | Vietnam | R-R | Rice | WS | 5 | 3 | 4 | 84.0 | 99.7 | 20.0 | 20.0 | 30.0 | 41.3 | 4.9 | 4.6 |  |  | 58.6 | 46.1 | 105 | 122 | 1229 | 1146 | 1125 | 1024 |
| ^29^ | India | R-U | Wheat |  | 46 |  |  | 153.0 | 145.5 | 24.2 | 24.6 | 66.4 | 5.8 | 4.8 | 4.0 |  |  | 31.4 | 27.1 | 179 | 136 | 960 | 790 | 781 | 654 |
| ^30^ | Indonesia | U-U | Maize | DS | 22 |  |  | 160.0 | 173.0 | 14.0 | 19.0 | 28.2 | 19.1 | 8.4 | 7.5 |  |  | 52.5 | 43.4 | 143 | 153 | 1260 | 1125 | 1117 | 972 |
|  | Philippines | U-U | Maize | DS | 31 |  |  | 132.0 | 107.0 | 15.0 | 12.0 | 24.1 | 14.9 | 9.1 | 7.5 |  |  | 68.9 | 70.1 | 124 | 97 | 1365 | 1125 | 1241 | 1028 |
|  |  |  |  |  |  |  |  |  |  |  |  |  |  |  |  |  |  |  |  |  |  |  |  |  |  |
| **Ref** | **Country** | **Cropping**  **system^ξ^** | **Crop** | **Season^ϑ^** | **Rep** | **No. of N splits** | | **N rate**  **(kg ha^-1^)** | | **P rate**  **(kg ha^-1^)** | | **K rate**  **(kg ha^-1^)** | | **Grain yield**  **(kg ha^-1^)** | | **AEN**  **(kg grain kg^-1^ N)** | | **PFP N**  **(kg grain kg^-1^ N)** | | **TFC**  **(USD ha^-1^)** | | **Gross return**  **(USD ha^-1^)** | | **GRF**  **(USD ha^-1^)** | |
|  |  |  |  |  |  | **SSNM** | **FFP** | **SSNM** | **FFP** | **SSNM** | **FFP** | **SSNM** | **FFP** | **SSNM** | **FFP** | **SSNM** | **FFP** | **SSNM** | **FFP** | **SSNM** | **FFP** | **SSNM** | **FFP** | **SSNM** | **FFP** |
| ^31^ | India | R-U | Rice |  | 323 |  |  | 111.0 | 85.0 | 14.8 | 17.0 | 40.7 | 39.0 | 5.8 | 4.6 |  |  | 52.1 | 54.5 | 120 | 106 | 1445 | 1158 | 1325 | 1051 |
|  | India | R-U | Maize |  | 412 |  |  | 145.0 | 151.0 | 20.1 | 27.5 | 53.1 | 35.7 | 6.7 | 5.4 |  |  | 45.9 | 35.6 | 158 | 163 | 998 | 807 | 839 | 644 |
|  | Philippines | R-U | Maize |  | 190 |  |  | 162.0 | 159.0 | 20.9 | 13.1 | 35.7 | 20.8 | 9.4 | 8.3 |  |  | 58.0 | 52.1 | 160 | 136 | 1409 | 1244 | 1249 | 1108 |
|  | China | R-U | Maize |  | 290 |  |  | 162.0 | 271.0 | 35.8 | 51.4 | 61.4 | 41.5 | 8.0 | 7.9 | 8.6 | 5.2 | 49.4 | 29.2 | 199 | 282 | 1200 | 1185 | 1001 | 903 |
|  | China | R-U | Rice |  | 137 |  |  | 156.0 | 170.0 | 30.5 | 25.7 | 72.2 | 70.6 | 8.0 | 7.8 | 15.0 | 12.2 | 51.3 | 45.9 | 194 | 194 | 2000 | 1950 | 1806 | 1756 |
|  | China | R-U | Wheat |  | 541 |  |  | 158.0 | 230.0 | 24.4 | 27.0 | 56.4 | 39.0 | 10.2 | 9.9 | 11.8 | 7.8 | 64.6 | 43.0 | 176 | 215 | 2040 | 1980 | 1864 | 1765 |
| ^32^ | India | U-U | Wheat | DS | 3 | 3 |  | 155.0 | 172.0 | 27.5 | 25.1 | 54.0 | 0.0 | 4.9 | 3.8 |  |  | 31.9 | 22.0 | 177 | 150 | 988 | 756 | 811 | 606 |
|  | India | U-U | Maize | WS | 3 | 3 |  | 170.0 | 110.0 | 16.1 | 13.1 | 36.5 | 0.0 | 4.5 | 3.8 |  |  | 26.7 | 34.3 | 158 | 91 | 682 | 567 | 524 | 475 |
| ^33^ | Vietnam | U-U | Maize |  | 10 |  |  | 155.0 | 188.5 | 43.3 | 49.8 | 90.5 | 89.8 | 7.5 | 6.8 | 28.3 | 18.5 | 51.0 | 36.6 | 225 | 256 | 1129 | 1013 | 904 | 756 |
|  | Philippines | U-U | Maize |  | 8 |  |  | 136.0 | 109.5 | 24.5 | 9.5 | 47.0 | 23.0 | 9.0 | 8.0 | 19.0 | 16.0 | 66.6 | 74.0 | 156 | 100 | 1350 | 1200 | 1194 | 1100 |
|  | Vietnam | R-U | Maize |  | 5 |  |  | 170.5 | 175.0 | 31.0 | 36.0 | 69.5 | 52.5 | 7.9 | 6.8 | 28.5 | 20.5 | 47.0 | 41.6 | 202 | 202 | 1178 | 1020 | 975 | 818 |
|  | Indonesia | R-U | Maize |  | 5 |  |  | 185.5 | 264.0 | 23.0 | 25.5 | 44.0 | 17.5 | 9.7 | 9.3 | 33.0 | 22.5 | 52.2 | 35.1 | 183 | 221 | 1448 | 1388 | 1264 | 1167 |
|  | Indonesia | U-U | Maize |  | 5 |  |  | 147.3 | 176.3 | 33.7 | 19.3 | 82.3 | 52.7 | 9.0 | 7.5 | 30.0 | 18.0 | 60.6 | 43.3 | 200 | 177 | 1345 | 1130 | 1145 | 953 |
| ^34^ | China | R-R | Rice | DS | 4 | 3 | 4 | 86.9 | 205.0 | 40.0 | 40.0 | 100.0 | 100.0 | 7.5 | 7.2 | 13.1 | 3.6 | 97.9 | 34.9 | 182 | 258 | 1886 | 1791 | 1704 | 1532 |
|  | Philippines | R-R | Rice | DS | 4 | 4 | 2 | 132.5 | 90.0 | 30.0 | 30.0 | 40.0 | 40.0 | 6.7 | 6.2 | 19.6 | 23.1 | 50.9 | 68.3 | 158 | 131 | 1663 | 1538 | 1505 | 1407 |
| ^35^ | Vietnam | R-R | Rice | DS | 24 | 4 |  | 101.5 | 106.3 | 23.0 | 18.0 | 65.5 | 19.5 | 5.7 | 5.3 | 22.0 | 17.0 | 56.3 | 49.9 | 143 | 109 | 1425 | 1325 | 1282 | 1216 |
|  | Vietnam | R-R | Rice | WS | 24 | 4 |  | 94.0 | 117.3 | 21.3 | 21.0 | 58.5 | 19.8 | 3.6 | 3.5 | 17.0 | 12.5 | 38.8 | 29.7 | 131 | 121 | 906 | 870 | 775 | 749 |
| ^36^ | India | R-U | Rice | WS | 3 | 3 |  | 118.0 | 130.0 | 11.8 | 17.4 | 43.2 | 16.6 | 6.4 | 6.1 | 24.1 | 19.4 | 54.1 | 46.6 | 122 | 122 | 1597 | 1516 | 1475 | 1394 |
|  | India | R-U | Rice | WS | 3 | 3 |  | 118.0 | 130.0 | 11.8 | 17.4 | 43.2 | 16.6 | 6.7 | 6.1 | 26.6 | 19.4 | 56.6 | 46.6 | 122 | 122 | 1669 | 1516 | 1547 | 1394 |
|  | India | R-U | Wheat | DS | 3 | 3 |  | 110.0 | 84.0 | 21.8 | 17.4 | 32.4 | 16.6 | 4.2 | 3.0 | 26.0 | 19.6 | 38.6 | 36.0 | 126 | 92 | 848 | 605 | 723 | 513 |
|  | India | R-U | Wheat | DS | 3 | 3 |  | 110.0 | 84.0 | 21.8 | 17.4 | 32.4 | 16.6 | 4.3 | 3.0 | 26.7 | 19.6 | 39.2 | 36.0 | 126 | 92 | 863 | 605 | 737 | 513 |
| ^37^ | India | R-R | Rice | DS | 15 | 3 |  | 120.0 |  | 15.0 |  | 50.0 |  | 6.6 | 5.9 |  |  | 54.6 |  | 139 |  | 1638 | 1475 | 1549 |  |
|  | India | R-R | Rice | WS | 15 | 4 |  | 122.5 |  | 15.0 |  | 50.0 |  | 6.2 | 5.8 |  |  | 50.4 |  | 140 |  | 1544 | 1438 | 1385 |  |
| ^38^ | Senegal |  | Rice | DS | 58 | 3 |  | 140.5 | 149.5 | 18.1 | 14.2 | 34.4 | 0.0 | 6.9 | 5.8 |  |  | 48.7 | 38.4 | 141 | 118 | 1713 | 1438 | 1572 | 1319 |
|  | Senegal |  | Rice | WS | 20 | 3 |  | 118.0 | 161.0 | 14.4 | 24.4 | 10.0 | 0.0 | 8.7 | 6.4 |  |  | 73.7 | 39.8 | 105 | 142 | 2175 | 1600 | 2070 | 1458 |
| ^39^ | India | U-U | Wheat |  | 30 |  |  |  |  |  |  |  |  | 5.5 | 4.7 |  |  |  |  |  |  | 1100 | 940 |  |  |
| ^40^ | India | R-U | Wheat | DS | 15 | 3 |  | 146.3 | 169.3 | 19.7 | 25.3 | 79.7 | 0.0 | 5.2 | 4.6 |  |  | 36.3 | 27.4 | 176 | 149 | 1045 | 929 | 869 | 780 |
| **Ref** | **Country** | **Cropping**  **system^ξ^** | **Crop** | **Season^ϑ^** | **Rep** | **No. of N splits** | | **N rate**  **(kg ha^-1^)** | | **P rate**  **(kg ha^-1^)** | | **K rate**  **(kg ha^-1^)** | | **Grain yield**  **(kg ha^-1^)** | | **AEN**  **(kg grain kg^-1^ N)** | | **PFP N**  **(kg grain kg^-1^ N)** | | **TFC**  **(USD ha^-1^)** | | **Gross return**  **(USD ha^-1^)** | | **GRF**  **(USD ha^-1^)** | |
|  |  |  |  |  |  | **SSNM** | **FFP** | **SSNM** | **FFP** | **SSNM** | **FFP** | **SSNM** | **FFP** | **SSNM** | **FFP** | **SSNM** | **FFP** | **SSNM** | **FFP** | **SSNM** | **FFP** | **SSNM** | **FFP** | **SSNM** | **FFP** |
| ^41^ | Thailand | R-R | Rice | DS | 24 | 4 |  | 118.0 | 121.8 | 17.1 | 24.0 | 35.5 | 4.3 | 4.6 | 4.7 | 8.9 | 10.5 | 39.0 | 38.9 | 125 | 119 | 1150 | 1175 | 1025 | 1056 |
|  | Thailand | R-R | Rice | WS | 24 | 4 |  | 106.3 | 102.3 | 18.8 | 21.3 | 50.8 | 6.0 | 5.0 | 4.8 | 8.4 | 6.5 | 47.3 | 47.2 | 130 | 103 | 1248 | 1188 | 1118 | 1084 |
| ^42^ | India | U-U | Maize | DS | 44 |  |  | 171.0 | 198.0 | 19.8 | 34.3 | 43.7 | 61.1 | 9.1 | 8.0 |  |  | 54.2 | 44.4 | 169 | 220 | 1368 | 1204 | 1199 | 983 |
|  | India | U-U | Maize | WS | 44 |  |  | 161.0 | 164.8 | 20.7 | 35.6 | 50.2 | 82.8 | 7.6 | 6.5 |  |  | 46.8 | 40.2 | 168 | 215 | 1135 | 971 | 967 | 756 |
| ^43^ | India | R-U | Maize | DS | 27 |  |  | 168.3 | 209.3 | 19.6 | 49.6 | 44.8 | 62.3 | 9.1 | 8.0 |  |  | 54.7 | 43.0 | 167 | 252 | 1366 | 1197 | 1198 | 945 |
|  | India | R-U | Maize | WS | 12 |  |  | 160.7 | 193.0 | 17.0 | 38.8 | 39.6 | 94.6 | 7.9 | 6.9 |  |  | 49.5 | 36.5 | 155 | 245 | 1190 | 1031 | 1035 | 786 |
| ^44^ | Burkina Faso | R-R | Rice | DS | 17 | 3 | 3 | 116.0 | 81.2 | 20.9 | 15.3 | 19.9 | 14.5 | 6.6 | 5.2 |  |  | 57.1 | 63.5 | 120 | 85 | 1656 | 1295 | 1536 | 1210 |
|  | Burkina Faso | R-R | Rice | WS | 13 | 3 | 3 | 116.0 | 76.2 | 20.9 | 16.9 | 19.9 | 16.1 | 6.3 | 5.2 |  |  | 53.9 | 68.3 | 120 | 86 | 1564 | 1306 | 1444 | 1221 |
| ^45^ | India | U-U | Maize | DS | 17 |  |  | 162.0 | 144.0 | 25.3 | 30.5 | 61.4 | 33.2 | 8.0 | 7.0 |  |  | 49.4 | 48.6 | 183 | 162 | 1200 | 1050 | 1017 | 888 |
| ^46^ | India | R-R | Rice | DS | 14 | 3 |  | 120.0 | 117.0 | 14.0 | 21.0 | 30.0 | 44.0 | 5.3 | 4.6 |  |  | 44.2 | 39.3 | 118 | 136 | 1325 | 1150 | 1207 | 1014 |
|  | India | R-R | Rice | WS | 40 | 3 |  | 130.0 | 124.5 | 14.0 | 21.0 | 41.5 | 46.5 | 4.6 | 4.2 |  |  | 35.4 | 33.7 | 132 | 143 | 1150 | 1038 | 1018 | 895 |
| ^47^ | India | R-R | Rice | DS | 19 | 3 |  | 99.5 | 96.6 | 12.9 | 25.5 | 24.0 | 45.1 | 5.1 | 4.7 |  |  | 50.9 | 48.5 | 99 | 131 | 1266 | 1172 | 1166 | 1041 |
|  | India | R-R | Rice | WS | 25 | 3 |  | 104.5 | 75.5 | 13.0 | 19.0 | 31.0 | 43.0 | 5.1 | 4.5 |  |  | 49.0 | 59.6 | 107 | 106 | 1280 | 1115 | 1173 | 1009 |
| ^48^ | India | R-U | Rice |  | 10 |  |  | 180.0 |  | 26.0 |  | 75.0 |  | 9.1 | 5.6 |  |  | 50.6 |  | 204 |  | 2278 | 1410 | 2073 |  |
|  | India | R-U | Wheat |  | 10 |  |  | 150.0 |  | 33.0 |  | 75.0 |  | 5.7 | 4.3 |  |  | 37.8 |  | 196 |  | 1134 | 866 | 938 |  |
| ^49^ | Vietnam | R-U | Rice | DS | 24 | 4 |  | 101.0 | 102.8 | 22.3 | 21.5 | 62.5 | 63.3 | 6.2 | 6.0 | 17.0 | 14.7 | 61.9 | 58.7 | 140 | 140 | 1550 | 1506 | 1410 | 1366 |
|  | Vietnam | R-U | Rice | WS | 24 | 4 |  | 86.3 | 105.0 | 10.0 | 18.3 | 43.3 | 60.0 | 6.2 | 6.0 | 18.0 | 13.5 | 71.2 | 57.2 | 99 | 134 | 1538 | 1500 | 1439 | 1366 |
| ^50^ | Vietnam | R-R | Rice | DS | 10 |  |  | 108.0 | 116.3 | 14.2 | 21.8 | 42.3 | 41.5 | 6.9 | 6.4 |  |  | 64.1 | 55.0 | 119 | 135 | 1718 | 1591 | 1599 | 1455 |
|  | Vietnam | R-R | Rice | WS | 10 |  |  | 90.0 | 108.7 | 20.6 | 20.3 | 37.9 | 38.5 | 4.7 | 4.5 |  |  | 53.6 | 42.1 | 114 | 126 | 1186 | 1133 | 1071 | 1006 |
| ^51^ | Bangladesh | R-R | Rice | DS | 20 |  |  |  |  |  |  |  |  | 6.0 | 5.8 | 29.0 | 22.0 |  |  |  |  | 1500 | 1450 |  |  |
|  | Bangladesh | R-R | Rice | WS | 8 |  |  |  |  |  |  |  |  | 4.2 | 4.2 | 23.0 | 12.0 |  |  |  |  | 1050 | 1050 |  |  |
|  | India | R-R | Rice |  | 48 |  |  | 67.9 | 91.6 |  |  |  |  | 5.7 | 5.5 |  |  | 98.7 | 75.9 |  |  | 1414 | 1385 |  |  |
| ^52^ | China | R-R | Rice | DS | 21 | 3 | 2 | 126.0 | 165.5 | 14.0 | 18.7 | 51.9 | 50.3 | 5.9 | 5.8 | 11.2 | 6.3 | 46.9 | 35.3 | 136 | 168 | 1467 | 1458 | 1331 | 1291 |
|  | China | R-R | Rice | WS | 21 | 3 | 2 | 126.0 | 177.3 | 13.5 | 20.0 | 51.9 | 64.3 | 7.1 | 6.6 | 13.6 | 7.0 | 57.1 | 37.1 | 135 | 186 | 1771 | 1638 | 1636 | 1451 |
| ^53^ | China | R-R | Rice | WS | 20 |  |  | 152.0 | 301.0 |  |  |  |  | 9.0 | 8.4 | 12.4 | 4.9 | 59.1 | 28.0 |  |  | 2245 | 2110 |  |  |
|  | China | R-R | Rice | DS and WS | 20 |  |  | 120.0 | 170.0 | 14.2 | 19.3 | 51.9 | 58.2 | 7.5 | 7.1 | 19.6 | 12.2 | 62.5 | 41.8 | 132 | 176 | 1875 | 1775 | 1743 | 1599 |
| ^54^ | China | R-R | Rice | DS | 21 | 3 | 2 | 130.0 | 170.0 | 22.5 | 22.5 | 75.0 | 90.0 | 5.8 | 5.4 | 11.4 | 6.3 | 44.6 | 31.8 | 167 | 202 | 1450 | 1350 | 1283 | 1148 |
|  | China | R-R | Rice | WS | 21 | 3 | 2 | 135.0 | 172.5 | 19.0 | 21.5 | 85.0 | 60.0 | 6.9 | 6.4 | 11.4 | 6.3 | 51.1 | 37.1 | 171 | 183 | 1725 | 1600 | 1554 | 1417 |
| **Ref** | **Country** | **Cropping**  **system^ξ^** | **Crop** | **Season^ϑ^** | **Rep** | **No. of N splits** | | **N rate**  **(kg ha^-1^)** | | **P rate**  **(kg ha^-1^)** | | **K rate**  **(kg ha^-1^)** | | **Grain yield**  **(kg ha^-1^)** | | **AEN**  **(kg grain kg^-1^ N)** | | **PFP N**  **(kg grain kg^-1^ N)** | | **TFC**  **(USD ha^-1^)** | | **Gross return**  **(USD ha^-1^)** | | **GRF**  **(USD ha^-1^)** | |
|  |  |  |  |  |  | **SSNM** | **FFP** | **SSNM** | **FFP** | **SSNM** | **FFP** | **SSNM** | **FFP** | **SSNM** | **FFP** | **SSNM** | **FFP** | **SSNM** | **FFP** | **SSNM** | **FFP** | **SSNM** | **FFP** | **SSNM** | **FFP** |
| ^55^ | China | R-R | Rice |  | 54 | 4 | 3 | 169.0 | 185.0 | 29.2 | 32.7 | 74.7 | 73.0 | 9.4 | 9.2 | 19.5 | 17.3 | 55.6 | 49.7 | 202 | 217 | 1410 | 1380 | 1208 | 1163 |
|  | China | R-U | Maize |  | 33 | 2 | 1 | 173.0 | 261.0 | 29.6 | 44.5 | 72.2 | 88.0 | 11.1 | 10.2 | 19.1 | 10.6 | 64.2 | 39.1 | 204 | 293,55 | 1665 | 1530 | 1461 | 1236 |
| ^56^ | China | R-R | Rice | WS | 10 | 3 | 2 | 102.0 | 150.0 | 39.2 |  | 62.3 |  | 6.5 | 6.6 |  | 16.6 | 64.1 | 43.7 | 167 |  | 1634 | 1638 | 1467 |  |
| ^57^ | China | U-U | Maize | DS | 55 | 2 | 2 | 157.9 | 225.9 | 23.7 | 27.0 | 54.4 | 38.7 | 10.3 | 10.1 | 11.9 | 7.9 | 65.1 | 46.0 | 173 | 212 | 1545 | 1520 | 1372 | 1308 |
| ^58^ | China | U-U | Maize | DS | 20 | 2 | 1 | 179.0 | 216.5 | 32.5 | 44.5 | 67.5 | 66.0 | 12.2 | 11.4 | 19.3 | 12.7 | 68.3 | 52.5 | 209 | 251 | 1835 | 1705 | 1626 | 1454 |
| ^59^ | China | R-R | Rice | DS | 10 | 3 |  | 151.5 | 203.5 | 27.3 | 30.3 | 78.0 | 92.5 | 7.6 | 7.0 | 16.0 | 12.5 | 49.9 | 34.4 | 190 | 237 | 1892 | 1750 | 1702 | 1513 |
|  | China | R-R | Rice | WS | 10 | 3 |  | 161.8 | 186.3 | 30.7 | 34.4 | 72.4 | 88.6 | 8.4 | 8.0 | 17.3 | 15.5 | 51.9 | 44.5 | 198 | 230 | 2104 | 2008 | 1906 | 1778 |
|  | China | R-U | Rice | WS | 10 | 3 |  | 149.0 | 189.0 | 31.6 | 36.0 | 79.3 | 80.9 | 9.0 | 8.4 | 16.0 | 9.5 | 60.2 | 44.3 | 196 | 229 | 2242 | 2092 | 2046 | 1862 |
| ^60^ | China | R-R | Rice | WS | 30 | 3 |  | 160.6 | 177.5 | 30.7 | 27.8 | 67.2 | 64.7 | 9.1 | 8.7 | 19.0 | 15.5 | 56.5 | 49.6 | 194 | 199 | 2263 | 2163 | 2068 | 1964 |
|  | China | R-R | Rice | DS and WS | 35 | 3 |  | 151.3 | 173.8 | 32.4 | 25.5 | 73.9 | 88.0 | 7.6 | 7.1 | 13.0 | 8.8 | 50.1 | 40.6 | 195 | 208 | 1900 | 1763 | 1705 | 1555 |
| ^61^ | China | U-U | Wheat | DS | 95 | 3 | 2 | 163.8 | 279.5 | 36.5 | 51.7 | 61.1 | 37.1 | 8.3 | 8.1 | 8.4 | 5.1 | 50.4 | 29.8 | 202 | 285 | 1650 | 1625 | 1448 | 1340 |

ξ for cropping system where R-R is rice-rice, R-U is rice-upland and U-U is upland-upland cropping system; ϑ for season where DS is the dry season and WS is the et season

**Supplementary Information Table 2: Categorical variables used in describing the experimental conditions**

| **Category** | **List** | **Description** |
| --- | --- | --- |
| **Region** Group of countries in a regional location where the study was conducted | East Asia | China |
|  | Southeast Asia | Indonesia, Philippines, Thailand, Vietnam |
|  | South Asia | Bangladesh, India, Nepal |
|  | Africa | Burkina Faso, Ghana, Senegal |
| **Cropping system** Refers to the long-term cultivation of crops and crop sequences in an agricultural field including all spatial and temporal aspects of managing an agricultural system | Rice-Rice | Continuous double- or triple-rice cropping |
|  | Rice-Upland | Rice-based cropping system where rice is grown in rotation with an upland crop (e.g. maize, wheat, pulses) |
|  | Upland-Upland | Upland crop in rotation with another upland crop |
| **Ecosystem** Refers to the agroecosystem based on soil-water conditions usually influenced by the climate in the area where the crops were grown | Irrigated | Water that is pumped directly from a source (groundwater, surface water or other bodies water) to the field and there are usually no major water shortages that negatively affect crop growth |
|  | Rainfed | Rainfall is the only source of water; crops may experience periods of draught stress |
| **Variety** Describes the breed or genetic makeup of the crop | Inbred | Pure variety where the succeeding offspring produced will have the same genes, resulting from a cross of 2 or more different varieties after several cycles of self-pollination |
|  | Hybrid | A product of a cross between two genetically distant parents, which usually results in a hybridization effect (e.g. more vigorous growth and higher yield) |
| **Season** Refers to the period of the year during which the particular crop is cultivated from the time of sowing to the time of harvesting | Dry season | Cropping season in the Tropics/Sub-Tropics with low rainfall. Dry season crops are usually grown under irrigation and yields tend to be higher because of greater solar radiation and less pest and diseases pressure. |
|  | Wet season | The part of the year when most of the rainfall occurs. Wet season crops can be rainfed or with partial irrigation. |
| **Residue** Crop residues such as stalks, stubble and leaves that are left in the field after the crop has been harvested | Removed | Crop residues removed from the field |
|  | Retained | Crop residues incorporated in the field by ploughing or other means. |
| **Crop Establishment** Describes the method of planting the crop | Transplanted | Seeds are sown in a nursery and seedlings are transferred to the field after 14-21 days |
|  | Direct-seeded | Seeds are sown directly to the field either manually or mechanically. |
| **Decision tool** The platform where a fertilizer recommendation is generated based on site-specific nutrient management (SSNM) approach. Initially developed for rice in the mid 1990s, the concept evolved to also cover other crops such as maize and wheat. Earlier decision tools for N includes leaf color charts (LCC) and the Soil Plant Analysis Development (SPAD) chlorophyll meter. Although they are still in-use, digital web-based decision tools such as the Rice Crop Manager (RCM), RiceAdvice and Nutrient Expert (NE) applications were developed in the 2000s and now serve as the main decision support tools for SSNM. | Only N is SSNM | Only N rate and timing is provided by SSNM recommendation; P and K are blanket-applied based on prevailing general fertilizer recommendations or farmers' practice |
|  | NPK is SSNM | SSNM recommendation for basal N, P and K rates was provided prior planting and N adjustments were done based on the LCC or SPAD-meter reading or N, P and K rates and timing was provided prior planting based from a farmers' actual interview or from his responses in a digital app. |
| **Farmer Fertilizer Practice Yield Category** Quantitatively estimated from the quartiles of the entire FFP grain yield data | Low | Grain yield values below 1^st^ quartile |
|  | Medium | Grain yield values between 1^st^ and 3^rd^ quartiles |
|  | High | Grain yield values above 3^rd^ quartile |
| **Type of trial** Describes the setting of the study | On-farm | The study was conducted in a farmers' field |
|  | On-station | The study was conducted in a research station |

**Supplementary Information Table 3: Correlation analysis (r^2^) for difference in parameters between site-specific nutrient management (SSNM) and farmer fertilizer practice (FFP); grain yield (∆GY), number of nitrogen (N) splits (∆N splits), N fertilizer rate (∆N rate), agronomic efficiency of N (∆AEN), partial factor productivity of N (∆PFP N), phosphorus fertilizer rate (∆P rate), potassium fertilizer rate (∆K rate), total fertilizer cost (∆TFC), gross return (∆Gross return), gross return above fertilizer cost (∆GRF)**

|  | ∆GY | ∆N splits | ∆N rate | ∆AEN | ∆PFP N | ∆P rate | ∆K rate | ∆TFC | ∆Gross  return | ∆GRF |
| --- | --- | --- | --- | --- | --- | --- | --- | --- | --- | --- |
| ∆GY | 1 | 0.29** | 0.35** | 0.45** | -0.20** | 0.22** | 0.17** | 0.39** | 0.96** | 0.80** |
| ∆N splits |  | 1 | 0.49** | ns | -0.49** | 0.17* | 0.44** | 0.58** | 0.24** | ns |
| ∆N rate |  |  | 1 | -0.44** | -0.78** | 0.36** | 0.17** | 0.75** | 0.36** | ns |
| ∆AEN |  |  |  | 1 | 0.50** | ns | ns | -0.29** | 0.41** | 0.54** |
| ∆PFP N |  |  |  |  | 1 | -0.13* | -0.13* | -0.55** | -0.20** | ns |
| ∆P rate |  |  |  |  |  | 1 | 0.38** | 0.67** | 0.29** | ns |
| ∆K rate |  |  |  |  |  |  | 1 | 0.63** | 0.18** | -0.11* |
| ∆TFC |  |  |  |  |  |  |  | 1 | 0.42** | ns |
| ∆Gross return |  |  |  |  |  |  |  |  | 1 | 0.84** |
| ∆GRF |  |  |  |  |  |  |  |  |  | 1 |

Statistical significance: ns = not significant; * <0.05; ** <0.01

**Supplementary Table 4: Previously published impact studies on site-specific nutrient management (SSNM) in Africa and Asia**

| **Reference** | **Crop** | **Country** | **DSS tool name** | **Approach** | **Yield increase** | **Profit increase** | **Remarks** |
| --- | --- | --- | --- | --- | --- | --- | --- |
| ^28^Pampolino et al. (2007) | Rice | Philippines | SSNM | Focus group discussions | 0.2 t/ha or 4% | 106 USD/ha/year or 10% | Double cropping of rice is primarily focused on profit, not yield. |
|  | Rice | India | SSNM | Focus group discussions | 0.8 t/ha or 17% | 168 USD/ha/year or 48% | The use of SSNM could result in reduced N_2_O emissions per unit of grain yield. |
|  | Rice | Vietnam | SSNM | Focus group discussions | 0.3 t/ha or 4% | 34 USD/ha/year or 4% |  |
| ^16^Islam et al. (2007) | Rice | India | Leaf color chart | “with and without” comparisons | 1 to 2% | 19 to 27 USD/ha | 17-21% N saved; reduced insecticide sprays by 50% |
| ^62^Rodriguez and Nga (2012) | Rice | Vietnam | SSNM | “with and without” comparisons | 0.6 t/ha or 11% | 150 USD/ha or 30% |  |
| ^63^Rejesus et al. (2014) | Rice | Bangladesh | Leaf color chart | “with and without” comparisons | - | 10 USD per capita income or 17% |  |
| ^64^Arouna et al. (2019) | Rice | Nigeria | RiceAdvice | Randomized control trial | 7% | 10% |  |
| ^65^Olyinbo et al. (2019) | Maize | Nigeria | Nutrient Expert | Randomized control trial | 0.2 to 0.3 t/ha or 10 to 14% | - |  |

**References**

1 Abdulrachman, S., Susanti, Z., Pahim, A. D., Dobermann, A. & Witt, C. Site-specific nutrient management in intensive irrigated rice systems of West Java, Indonesia. *Increasing Productivity of Intensive Rice Systems Through Site-Specific Nutrient Management* **2004**, 171-192 (2004).

2 Center, A. R. Terminal report on collaboration project between Syngenta Foundation for Sustainable Agriculture (SFSA) and Africa Rice Center (AfricaRice): validating the services of a decision support system for nutrient management for rice in West Africa. (2016).

3 Alam, M. M., Ladha, J., Khan, S. R., Khan, A. & Buresh, R. Leaf color chart for managing nitrogen fertilizer in lowland rice in Bangladesh. *Agronomy Journal* **97**, 949-959 (2005).

4 Alam, M. M. *et al.* Nutrient management for increased productivity of rice–wheat cropping system in Bangladesh. *Field crops research* **96**, 374-386 (2006).

5 Banayo, N. P., Haefele, S. M., Desamero, N. V. & Kato, Y. On-farm assessment of site-specific nutrient management for rainfed lowland rice in the Philippines. *Field Crops Research* **220**, 88-96 (2018).

6 Bhende, S. N. & Kumar, A. Nutrient Expert®-based fertiliser recommendation improved wheat yield and farm profitability in the Mewat. *Better Crops-South Asia* **8**, 21-23 (2014).

7 Singh, B. Site specific and need based management of nitrogen fertilizers in cereals in India. *Advances in Fertilizer Technology: Biofertilizers* **2**, 576-605 (2014).

8 Biradar, D., Aladakatti, Y., Rao, T. & Tiwari, K. Site-specific nutrient management for maximization of crop yields in Northern Karnataka. *Better Crops* **90**, 33-35 (2006).

9 Budhathoki, S., Amgain, L. P. & Subedi, S. Assessing Growth, Productivity and Profitability of Drought Tolerant Rice Using Nutrient Expert-Rice and Other Precision Fertilizer Management Practices in Lamjung, Nepal.

10 Chuan, L. *et al.* Establishing a scientific basis for fertilizer recommendations for wheat in China: Yield response and agronomic efficiency. *Field Crops Research* **140**, 1-8 (2013).

11 Dahal, S., Shrestha, A., Dahal, S. & Amgain, L. P. Nutrient Expert Impact on Yield and Economic In Maize and Wheat. *International Journal of Applied Sciences and Biotechnology* **6**, 45-52 (2018).

12 Dutta, S. K. *et al.* Nutrient Expert-wheat: a tool for increasing crop yields and farm profit. *Better Crops-South Asia* **8**, 11-13 (2014).

13 Gines, H., Redondo, G., Estigoy, A. & Dobermann, A. Site-specific nutrient management in irrigated rice systems of Central Luzon, Philippines. *Increasing Productivity of Intensive Rice Systems through Site-Specific Nutrient Management. Science Publishers, Enfield, NH (USA) and International Rice Research Institute, Los Banos, Philippines*, 145-169 (2004).

14 Gupta, G., Shrestha, A., Shrestha, A. & Amgain, L. Evaluation of different nutrient management practice in yield and growth in rice in Morang district. *Adv Plants Agric Res* **3**, 187-191 (2016).

15 Hu, R. *et al.* Farmer participatory testing of standard and modified site-specific nitrogen management for irrigated rice in China. *Agricultural Systems* **94**, 331-340 (2007).

16 Islam, Z., Bagchi, B. & Hossain, M. Adoption of leaf color chart for nitrogen use efficiency in rice: Impact assessment of a farmer-participatory experiment in West Bengal, India. *Field Crops Research* **103**, 70-75 (2007).

17 Jat, R. *et al.* Conservation agriculture and precision nutrient management practices in maize-wheat system: effects on crop and water productivity and economic profitability. *Field Crops Research* **222**, 111-120 (2018).

18 Khanal, S., Dhakal, B., Bhusal, K. & Amgain, L. P. Assessment of Yield and Yield Attributing Characters of Hybrid Maize using Nutrient Expert® Maize Model in Eastern Terai of Nepal. *International Journal of Environment, Agriculture and Biotechnology* **2**, 238956 (2017).

19 Khuong, T. Q., Thi, T., Huan, N., Tan, P. & Buresh, R. Effect of site specific nutrient management on grain yield, nutrient use efficiency and rice production profit in the Mekong Delta. *Omonrice* **158**, 153-158 (2007).

20 Khurana, H. S., Phillips, S. B., Dobermann, A., Sidhu, A. S. & Peng, S. Performance of site‐specific nutrient management for irrigated, transplanted rice in northwest India. *Agronomy journal* **99**, 1436-1447 (2007).

21 Khurana, H. S. *et al.* Agronomic and economic evaluation of site-specific nutrient management for irrigated wheat in northwest India. *Nutrient Cycling in Agroecosystems* **82**, 15-31 (2008).

22 Khurana, H. S., Sidhu, A., Singh, B. & Singh, Y. Performance of Site-Specific Nutrient Management in a Rice-Wheat Cropping System. The Proceedings of the International Plant Nutrition Colloqium XVI, UC Davis. <https://escholarship.org/uc/item/870498gc>. (2009).

23 Kumar, A. *et al.* Evaluation of nutrient Expert TM for wheat. *Better Crops-South Asia* **6**, 27-29 (2012).

24 Majumdar, K. *et al.* On-farm performance of „Nutrient Expert‟ for maize: fertilizer recommendation, yield and nutrient use efficiency. *Better Crops South-Asia* **8**, 24-27 (2014).

25 Mandal, M. *et al.* Enhancing Rice Yield, Profitability, and Phosphorus Use Efficiency in West Bengal using the Nutrient Expert® Fertilizer Decision Support Tool. *Better Crops–South Asia*, 12 (2015).

26 Marahatta, S. Increasing productivity of an intensive rice based system through site specific nutrient management in Western Terai of Nepal. *Journal of Agriculture and Environment* **18**, 140-150 (2017).

27 Nagarajan, R. *et al.* Site-specific nutrient management in irrigated rice systems of Tamil Nadu, India. *Increasing Productivity of Intensive Rice Systems through Site-Specific Nutrient Management. Science Publishers, Enfield, NH (USA) and International Rice Research Institute, Los Banos, Philippines*, 101-123 (2004).

28 Pampolino, M. F. *et al.* Environmental impact and economic benefits of site-specific nutrient management (SSNM) in irrigated rice systems. *Agricultural Systems* **93**, 1-24 (2007).

29 Pampolino, M. F., Witt, C., Pasuquin, J. M., Johnston, A. & Fisher, M. J. Development approach and evaluation of the Nutrient Expert software for nutrient management in cereal crops. *Computers and electronics in agriculture* **88**, 103-110 (2012).

30 Pampolino, M. F., Witt, C., Pasuquin, J. M., Johnston, A. M. & Fisher, M. J. Development and evaluation of Nutrient Expert® decision support tool for cereal crops. *Better Crops-South Asia* **8**, 4-6 (2014).

31 Pampolino, M. Optimising fertilizer formulations for smallholders in Asia and Africa. *Fertilizer Focus*, 42-45 (2016).

32 Parihar, C. *et al.* Effects of precision conservation agriculture in a maize-wheat-mungbean rotation on crop yield, water-use and radiation conversion under a semiarid agro-ecosystem. *Agricultural Water Management* **192**, 306-319 (2017).

33 Pasuquin, J. *et al.* Closing yield gaps in maize production in Southeast Asia through site-specific nutrient management. *Field Crops Research* **156**, 219-230 (2014).

34 Peng, S. *et al.* Strategies for overcoming low agronomic nitrogen use efficiency in irrigated rice systems in China. *Field Crops Research* **96**, 37-47 (2006).

35 Tan, P. S. *et al.* Site-specific nutrient management in irrigated rice systems of the Mekong Delta of Vietnam. *Increasing productivity of intensive rice systems through site-specific nutrient management. Science, Inc., Enfield*, 193-215 (2004).

36 Qureshi, A., Singh, D. K. & Kumar, A. Climate Smart Nutrient Management (CSNM) for Enhanced Use Efficiency and Productivity in Rice and Wheat under Rice-Wheat Cropping System. *International Journal of Current Microbiology and Applied Sciences* **7**, 4166-4176 (2018).

37 Rajendran, R., Stalin, P., Ramanathan, S. & Buresh, R. Site-Specific Nitrogen and Potassium Management for Irrigated Rice in the Cauvery Delta. *Better Crops*, 7 (2010).

38 Saito, K., Diack, S., Dieng, I. & N’Diaye, M. K. On-farm testing of a nutrient management decision-support tool for rice in the Senegal River valley. *Computers and Electronics in Agriculture* **116**, 36-44 (2015).

39 Sapkota, T. B. *et al.* Precision nutrient management in conservation agriculture based wheat production of Northwest India: Profitability, nutrient use efficiency and environmental footprint. *Field Crops Research* **155**, 233-244 (2014).

40 Sapkota, T. B., Majumdar, K. & Jat, M. Precision nutrient management in no-till wheat: A case study for Haryana. *BETTER CROPS–SOUTH ASIA*, 16 (2014).

41 Satawathananont, S. *et al.* Site-specific nutrient management in irrigated rice systems of Central Thailand. *Increasing productivity of intensive rice systems through site-specific nutrient management. International Rice Research Institute, Los Baños*, 125-143 (2004).

42 Satyanarayana, T. *et al.* Nutrient Expert®–Maize: A Tool for Increasing Crop Yields and Farm Profit. *Better Crops* **8**, 7-10 (2014).

43 Satyanarayana, T. *et al.* Nutrient ExpertTM: A Tool to Optimize Nutrient Use and Improve Productivity of Maize. *Better Crops* **97**, 21-24 (2013).

44 Segda, Z., Haefele, S. M., Wopereis, M. C. S., Sedogo, M. P. & Guinko, S. Combining Field and Simulation Studies to Improve Fertilizer Recommendations for Irrigated Rice in Burkina Faso. *Agronomy Journal* **97**, 1429-1437, doi:10.2134/agronj2004.0275 (2005).

45 Shahi, V., Dutta, S., Majumdar, K., Satyanarayana, T. & Johnston, A. Nutrient Expert® Improves Maize Yields while Balancing Fertiliser Use. *Better Crops* **8**, 14-15 (2014).

46 Sharma, S. *et al.* Web-based tool for calculating field-specific nutrient management for rice in India. *Nutrient Cycling in Agroecosystems* **113**, 21-33, doi:10.1007/s10705-018-9959-x (2019).

47 Sharma, S. *et al.* Field-specific nutrient management using Rice Crop Manager decision support tool in Odisha, India. *Field crops research* **241**, 107578 (2019).

48 Singh, V. *et al.* Effect of site-specific nutrient management on yield, profit and apparent nutrient balance under pre-dominant cropping systems of Upper Gangetic Plains. *Indian Journal of Agricultural Sciences* **85**, 335-343 (2015).

49 Son, T. T., Chien, N. V., Thoa, V. T. K., Dobermann, A. & Witt, C. Site-specific nutrient management in irrigated rice systems of the Red River Delta of Vietnam. Increasing productivity of intensive rice systems through site-specific nutrient management. Science, Inc., Enfield. (2004).

50 Van Hach, C. & Tan, P. S. Study on site-specific nutrient management (SSNM) for high-yielding rice in the Mekong Delta. *Omonrice* **15**, 144-152 (2007).

51 Varinderpal, S. *et al.* Site-specific fertilizer nitrogen management for timely sown irrigated wheat (Triticum aestivum L. and Triticum turgidum L. ssp. durum) genotypes. *Nutrient Cycling in Agroecosystems* **109**, 1-16, doi:10.1007/s10705-017-9860-z (2017).

52 Wang, G. *et al.* Site-specific nutrient management in irrigated rice systems of Zhejiang Province, China. Increasing productivity of intensive rice systems through site-specific nutrient management. Science, Inc., Enfield. (2004).

53 Wang, G., Zhang, Q., Witt, C. & Buresh, R. Opportunities for yield increases and environmental benefits through site-specific nutrient management in rice systems of Zhejiang province, China. *Agricultural Systems* **94**, 801-806 (2007).

54 Wang, G., Dobermann, A., Witt, C., Sun, Q. & Fu, R. Performance of site‐specific nutrient management for irrigated rice in southeast China. *Agronomy Journal* **93**, 869-878 (2001).

55 Wang, Y. *et al.* Agronomic and environmental benefits of nutrient expert on maize and rice in Northeast China. *Environmental Science and Pollution Research International* (2020).

56 Xu, Y. *et al.* Agronomic performance of late-season rice under different tillage, straw, and nitrogen management. *Field Crops Research* **115**, 79-84, doi:<https://doi.org/10.1016/j.fcr.2009.10.005> (2010).

57 Xu, X. *et al.* Fertilizer recommendation for maize in China based on yield response and agronomic efficiency. *Field Crops Research* **157**, 27-34, doi:<https://doi.org/10.1016/j.fcr.2013.12.013> (2014).

58 Xu, X. *et al.* Narrowing yield gaps and increasing nutrient use efficiencies using the Nutrient Expert system for maize in Northeast China. *Field Crops Research* **194**, 75-82, doi:<https://doi.org/10.1016/j.fcr.2016.05.005> (2016).

59 Xu, X. *et al.* Methodology of fertilizer recommendation based on yield response and agronomic efficiency for rice in China. *Field Crops Research* **206**, 33-42, doi:<https://doi.org/10.1016/j.fcr.2017.02.011> (2017).

60 Yang, F. *et al.* Experimental validation of a new approach for rice fertiliser recommendations across smallholder farms in China. *Soil Research* **55**, 579-589, doi:<https://doi.org/10.1071/SR16328> (2017).

61 Zhang, J. *et al.* Nutrient expert improves nitrogen efficiency and environmental benefits for winter wheat in China. *Agronomy Journal* **110**, 696-706 (2018).

62 Rodriguez, D. G. P. & Nga, N. T. D. Impacts of site-specific nutrient management in irrigated rice farms in the Red River Delta, Northern Vietnam. (2012).

63 Rejesus, R. M., Martin, A. M. & Gypmantasiri, P. Enhancing the impact of natural resource management research: Lessons from a meta-impact assessment of the Irrigated Rice Research Consortium. *Global Food Security* **3**, 41-48 (2014).

64 Arounaa, A., Michlerb, J. D., Yergoa, W. G. & Saitoa, K. One Size Does Not Fit All: Experimental Evidence on the Digital Delivery of Personalized Extension Advice in Nigeria. (2019).

65 Oyinbo, O. *et al.* Can site-specific extension services improve fertilizer use and yields? Experimental evidence from Nigeria. (2019).
